# Role of Photobiomodulation Therapy in Modulating Oxidative Stress in Temporomandibular Disorders. A Systematic Review and Meta-Analysis of Human Randomised Controlled Trials

**DOI:** 10.3390/antiox10071028

**Published:** 2021-06-25

**Authors:** Reem Hanna, Snehal Dalvi, René Jean Bensadoun, Stefano Benedicenti

**Affiliations:** 1 Department of Surgical Sciences and Integrated Diagnostics, Laser Therapy Centre, University of Genoa, Viale Benedetto XV 6, 16132 Genoa, Italy; stefano.benedicenti@unige.it; 2 Department of Oral Surgery, Dental Institute, King’s College Hospital NHS Foundation Trust, London SE5 9RS, UK; 3 Department of Periodontology, Swargiya Dadasaheb Kalmegh Smruti Dental College and Hospital, Nagpur 441110, India; drsnehaldeotale@gmail.com; 4 Department of Oncology Radiology, Centre De Haute Energie, 10 Boulevard Pasteur, 06000 Nice, France; renejean.bensadoun@che-nice.com

**Keywords:** low-level laser therapy, oxidative stress, reactive oxygen species, temporomandibular joint disorder, randomised controlled trials, photobiomodulation, light-emitting diodes, orofacial pain, TMD standard care, synovial joint proinflammatory mediators

## Abstract

This systematic review and meta-analysis (PROSPERO registration; ref CRD 42020198921) aimed to govern photobiomodulation therapy (PBMT) efficacy in temporomandibular disorder (TMD). PRISMA guidelines and Cochrane Collaboration recommendations were followed. Differences in pain reduction assessment by qualitative measurement with visual analogue scale (VAS), pain pressure threshold (PPT) and maximum mouth opening (MMO) were calculated with 95% confidence intervals and pooled in a random effects model with a subgroup analysis, evaluating the role of follow-up duration. Heterogeneity was analysed using Q and I^2^ tests. Publication bias was assessed by visual examination of funnel plot symmetry. Qualitative analysis revealed 46% of the 44 included studies showed a high risk of bias. Meta-analysis on 32 out of 44 studies revealed statistically significant intergroup differences (SSID) for VAS (SMD = −0.55; 95% CI = −0.82 to −0.27; Z = 3.90 (*p* < 0.001)), PPT (SMD = −0.45; 95% CI = −0.89 to 0.00; Z = 1.97 (*p* = 0.05)) and MMO (SMD = −0.45; 95% CI = −0.89 to 0.00; Z = 1.97 (*p* = 0.05)), favouring PBMT compared to control treatment strategies. Sensitivity analysis revealed SSID (SMD = −0.53; 95% CI = −0.73 to −0.32; Z = 5.02 (*p* < 0.0001)) with low heterogeneity (Τ^2^ = 0.02; χ^2^ = 16.03 (*p* = 0.31); I^2^ = 13%). Hence, this review, for first time, proposed suggested recommendations for PBMT protocols and methodology for future extensive TMD research.

## 1. Introduction

TMD is considered as one of the main triggers in inducing orofacial pain of non-dental origin, which can have a negative impact on a patient’s functionality and psychological status [1,2], which ultimately can affect quality of life (QoL) [3]. Hence, the International Association for the Study of Pain (IASP) has defined TMD as a set of clinical conditions with signs and symptoms in the masticatory muscles, temporomandibular joint (TMJ) and associated structures (fatigue or stiffness of the jaws) and pain on palpation of the masticatory muscles [4].

**Figure 1:**
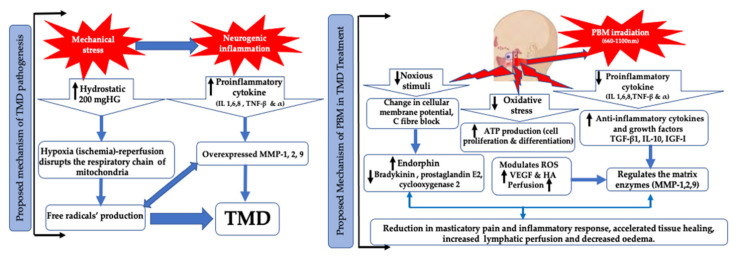
Schematic representation of the proposed aetiopathology mechanism of TMD and PBM mechanism of action in TMD management. Abbreviations: IL: Interleukin; TNF-α & β: transforming necrosis factor-beta and alpha; ROS: reactive oxygen species; ATP: adenosine triphosphate; MMP-1,2,9: matrix metalloproteinases-1,2,9; VEGF: vascular endothelial growth factor; HA: hyaluronic acid. All the abbreviations in this table are listed in Supplementary File S2.

The pathogenesis and aetiology of TMD are complex and not clearly understood. The production of free radicals, proinflammatory cytokines, nociceptive neuropeptides [5] and growth factors can lead to inflammation, pain and progressive tissue changes [6]. Due to the complexity of TMD pathophysiological, several treatment modalities have been implemented in TMD management, such as acupuncture, occlusal splints [7], kinesiotherapy, therapeutic jaw exercises [8], physiotherapy, postural training, psychotherapy, pharmacotherapy [9], transcutaneous electrical nerve stimulation (TENS) [10] and behavioural therapies. However, due to the diversity in the efficacy of these therapies, they remain unable to provide long-term relief of the associated symptoms and restoration of the mandibular functions [11]. Understanding TMD pathogenesis is crucial in order to prescribe an effective therapy to achieve optimal outcomes. In TMD, reactive oxygen species (ROS) can be generated via various pathways, such as direct mechanical injury, hypoxia–reperfusion and arachidonic acid catabolism to the articular tissues [12]. There is a lack of full understanding of the mechanical stresses generated during human jaw movements [13] (Figure 1). The vital role of oxidative stress (OS) in TMD pathogenesis is related to OS level imbalance, resulting in changes in the synovial fluid composition, such as alterations in viscosity, molecular size and cytokine levels [14]. Additionally, it has a part in disease onset and or progression, such as degradation of cartilage and sub-chondral bone, intra-articular damage, inflammation, pain and restricting range of motion, resulting in a negative QoL [14]. Moreover, stiffness of TMJ condyle cartilage is related to lack of collagen elasticity due to ageing [15]. There is strong evidence of overexpression of MMP-1, MMP-2 and MMP-9 in the synovial fluid of TMD patients [16,17].

Photobiomodulation (PBM) (laser or light-emitted diodes (LEDs)) therapy has gained interest as a non-invasive tool with immunomodulatory, anti-inflammatory and bioregenerative effects in stimulating healing, relieving pain and reducing inflammation [18,19]. The light of the optical window between 600 and 1200 nm is absorbed by cells’ chromophores. The cytochrome c oxidase (CCO) of mitochondrial electron chain transport complex IV absorbs photonic energy of wavelengths from 600 to 900 nm, whereas longer wavelengths are understood to be absorbed by water and light-sensitive ion channels. PBM can also reduce the oxidative stress by dissociating the inhibitory nitric oxide (NO) from CCO [20]. PBM modulates OS and reactive oxygen species (ROS)-mediated signalling in TMD management. PBM laser therapy is proven to enhance perfusion to bone and joint structures, stimulate osteoblasts [21] and chondrocytes, reduce the inflammatory cytokines and chemokines, increase anti-inflammatory cytokines and reduce nerve stimulation [21,22], promote endogenous opioids release, enhance tissue healing, increase angiogenesis, augment muscle tensile strength [23], increase the pain threshold by affecting the cellular membrane potential and decrease inflammation, possibly due to the reduction in prostaglandin E2 and suppression of cyclooxygenase 2 (COX-2) levels [24,25] (Figure 1). Moreover, PBM enhances the lymphatic system response [26].

Utilisation of PBM therapy (PBMT) for TMD management can be an effective treatment in modulating pain intensity by reducing inflammation while exhibiting regenerative and analgesic effects [27,28]. However, its clinical efficacy has been controversial due to the diversity of the reported results. Many systematic reviews and meta-analyses have analysed PBMT effectiveness in musculoskeletal disorders [29,30], but only three relatively focused on TMDs in which effective dosage and power density were analysed to evaluate the reduction in TMD-induced pain [31,32,33]. Additionally, the use of LED PBM has captured the interest of many investigators, due to its easy administration over a large surface area [34]. Hence, it is significantly important to explore its use for TMD, as different light sources and intensities might produce different or better results [35,36]. As little light has been shed on this matter, it was included in the present review. Clinicians, scholars and investigators have collectively concluded within the current available RCTs that further robust double-blind RCTs with vigorous standardised methodology and laser dosimetry are needed.

The rationale of conducting the present systematic review and meta-analysis was in the line of the above-mentioned key factors. 

The present systematic review and meta-analysis aimed to evaluate the existing contextual scientific evidence, justifying the gaps in the literature and building a conceptual framework to govern the efficacy of PBMT in TMD management. 

The objectives of this research review are listed below:

1. To explore the basis of and extrapolate the reasons for the inconsistencies among the data.
2. To evaluate the sensitivity of the results’ methods of assessment and obtain vigorous standardised methodology.
3. To attempt to propose a preliminary empirical consensus of PBM laser and LED dosimetry and treatment protocols.
4. To postulate extraoral (EO) and intraoral (IO) treatment strategies for TMD for future randomised clinical trial (RCT) studies.

The highlights of this research review are listed below:

1. The pathogenesis and aetiology of temporomandibular disorder (TMD) are complex and not clearly understood.
2. Oxidative stress and reactive oxygen species play a vital part in TMD pathogenesis and its progression.
3. Photobiomodulation (PBM) therapy is an effective treatment modality, as mono-therapy of various light sources of single wavelength or in combination of two wavelengths, in improving chronic pain, functionality, anxiety/depression and, subsequently, quality of life in patients with TMD.
4. This review, for the first time, addressed the standardisation of methodology and PBM protocols by proposing suggested recommendations, which can only be used to pave the roadmap for future extensive research in the management of TMD chronic symptoms.

## 2. Materials and Methods

### 2.1. Protocol and PROSPERO Registration

This systematic review was conducted in accordance with the guidelines of the Preferred Reporting Items for Systematic Reviews and Meta-Analysis (PRISMA) statement and Cochrane Handbook for Systematic Reviews (Supplementary File S1) [37,38]. Review protocol is published in Prospective Register Of Systematic Reviews (PROSPERO); ref CRD 42020198921.

### 2.2. Population (P), Intervention (I), Comparison (C) and Outcomes (O)—PICO

- **P:** Subjects with diagnostic criteria for temporomandibular disorder (RDC/TMD) [38,39,40].
- **I:** Effect of PBMT with light-emitting diodes (LEDs) or laser on TMD, including chronic pain, masticatory malfunction, anxiety/depression and quality of life (QoL).
- **C:** Placebo (sham PBM), pharmacological approach, cognitive approach, physiotherapy, conservative treatment modalities (occlusal splint), ultrasound, TENS, alpha lipoic acid, needle therapy or combined therapy (PBM and any standard care treatment).
- **O:** Pain intensity (PI) reduction, functional enhancement, anxiety/depression improvement or QoL improvement.

### 2.3. Focused Questions of Review Search

The capsulised focused research review questions were as follows:

1. Does PBM with laser or LEDs or combined therapies have superior effects compared to placebo or TMD standard care, or combined therapies (PBMT and standard care), in reducing pain intensity or improving patients’ functionality and psychological status, as well as QoL?
2. Does combined laser PBM therapy of red and IR wavelengths provide synergistic effects compared to placebo?
3. Is it possible to propose clinical guidance and recommendations of PBMT (LEDs and laser) for TMD management?

### 2.4. Search Strategy

The search strategy included only terms related to or describing the study domain and intervention, which were conducted by two review authors (R.H. and S.D.) independently. In order to assess inter-reviewer reliability, Kappa (κ) statistics were performed, with a minimum value of 0.8 deemed acceptable [41]. In the case of any inconsistencies, a third review author (S.B.) was consulted to resolve the matter. The following databases, using the relevant keywords and Medical Subjective Headings (MeSH) Terms, were systematically searched: MEDLINE (NCBI PubMed and PMC), EMBASE, CINAHL, ClinicalTrials.gov, the Cochrane Library database, ProQuest, Scopus, Trial Registry for RCTs, comparing PMBT with a placebo or standard care intervention or combined therapies in patients with TMD, Cochrane Central Register of Controlled Trials (CCRCT), ScienceDirect and Google Scholar. Additionally, the following journals were hand searched: Photomedicine and Laser Surgery, The Journal of Headache and Pain, Journal of Biophotonics, Journal of Dental Research, Lasers in Medical Science, Journal of Photochemistry and Photobiology, Pain Journal, Journal of Orofacial Pain, Medicine, J. Phys. Therapy. Sci., BMJ Open, J Craniofac. Surg., Journal of Neuroscience, Nature Neuroscience, J Craniomandibular Disord., Occupational Therapy International, Oral Diseases, Clin J Pain, Laser Ther. and Oral Surg Oral Med Oral Pathol Oral Radiol Endod. The electronic search was thoroughly explored during the period 1 January 2005—31 January 2021. 

### 2.5. Relevant Free Keywords and MeSH Terms

The following keyword search terms were used to capture the relevant domain and interventions of RCT study:

“Temporomandibular disorder(s)” **OR** “Temporomandibular joint disorder(s)” **OR** “Temporomandibular joint dysfunction” **OR** “TMJ disorder (s)” **OR** “TM disorder(s)” **OR** “Temporomandibular joint pain” **OR** “Temporomandibular pain” **OR** “TM pain” **OR** “TMJ pain” **OR** “TMD” **OR** “Myofascial pain” **OR** “Craniomandibular disorder(s)” **OR** “Mandibular dysfunction” **OR** “Osteoarthritis” **OR** “Temporomandibular joint dysfunction syndrome”.

**AND**

“Laser” **OR** “laser therapy” **OR** “Phototherapy” **OR** “low level laser therapy” **OR** “low energy laser therapy (LELT)” **OR** “LLLT” **OR** “infrared (IR) laser” **OR** “IR laser” **OR** “Light emitted diodes” **OR** ”LEDs” **OR** “Gallium-arsenide laser” **OR** “Gallium-aluminium-arsenide laser” **OR** “GaAlAs laser”.

**AND**

“Physical therapeutic agents” **OR** “TENS” **OR** “Transcutaneous electric nerve stimulation” **OR** “Medications” **OR** Physiotherapy” **OR** “Jaw exercises” **OR** “Occlusal split” OR “QoL” **OR** “Quality of life” **OR** “Pain” **OR** “Masticatory pain” **OR** “Anxiety” OR “Mandibular movement”.

**AND**

“Randomised Controlled Trials” **OR** “RCTs”.

Each of the below MeSH Terms was used to find the relevant literature from the search engines in Section 2.4:

“Temporomandibular Joint Disorders/LED” (Mesh) **OR** “Temporomandibular Joint Disorders/LLLT” (Mesh) **OR** “Temporomandibular Joint Disorders/laser” (Mesh)) **AND** “Humans” (Mesh) **AND** “Immunological profile” (Mesh) **AND** “orofacial pain” (Mesh).

“Temporomandibular Joint Disorders” (MeSH Major Topic) **AND** “Myofascial pain” [MeSH Major Topic]) **AND** “Humans” (MeSH) **AND** “LLLT” (MeSH Major Topic) **OR** “Photobiomodulation” (MeSH Major Topic) **OR** “PBM therapy” (MeSH Major Topic).

### 2.6. Eligibility Criteria

#### 2.6.1. Inclusion Criteria

1. Both genders of mean age of ≥18 years old, diagnosed with TMD by the degrees of this dysfunction based on the Research Diagnostic Criteria for Temporomandibular Disorder (RDC/TMD) [38,39].
2. Studies of in vivo human randomised controlled trials (RCTs) (split-mouth, parallel or prospective), comparing the efficacy of PBMT to any other standard care treatment modalities or combined therapies (PBM and one standard care treatment).
3. Studies investigating the effects of PBMT on TMD symptoms; chronic pain for ≥3 months in TMJ or masticatory muscles, loss of movement or masticatory malfunction for at least 3 months were included.
4. Light sources: laser or light-emitting diodes (LEDs) with no wavelength restrictions.
5. Studies reporting at least one of the following parameters as an outcome variable: pain score, functionality score, qualify of life or immunological profile.
6. Studies reporting any of the following outcomes: immediately after treatment, middle of treatment and end of treatment.
7. Studies reporting any follow-up timepoint: short-term, >2 weeks and <2 months; intermediate-term, ranging between >2 months and <6 months; and long-term, >6 months.
8. If multiple terms of outcomes were reported within one period, a period the closest to two weeks, one month, three months and six months for each follow-up timepoint respectively used.
9. All timepoints assessments of additional outcomes (all): baseline, immediate post treatment, short-term, intermediate-term and long-term follow-up.
10. No language restrictions for search strategy.
11. No restrictions on the reported laser parameters.
12. Subjects with one or more of the following symptoms: mandibular activities aggravate pain and functional disabilities, pain clicking, mandibular movements (MM) limitation or myogenous or arthrogenous TMJ pain.
13. Data search was during the period 1 January 2005–31 January 2021.

#### 2.6.2. Exclusion Criteria

1. Studies utilised home or stellate ganglion or acupuncture PBM (laser or LEDs) approach.
2. Other neuropathic orofacial pain conditions not related to TMD.
3. Studies utilised pharmacotherapy as a primary outcome.
4. Studies utilised a combined physiotherapeutic, pharmacotherapy and homeopathic measure.
5. Physiological or systematic conditions contributing to the pain.
6. Subjects with the following systemic diseases: cardiovascular, infection, inflammatory, neurological, metabolic, rheumatoid, osteoarthritis (changes in the fossa and condyle), autoimmune disorders.
7. Subjects with mental illnesses which could affect the clinical picture of patients, cervical disc herniation, history of trauma, TMJ surgery, musculo-articular pathologies, history of facial trauma, TMJ disc or condyle erosion, fibromyalgia, removable denture, missing more than one tooth in each quadrant and major malocclusion (anterior open bite, maxillary unilateral lingual cross-bite and overjet greater than 6 mm).
8. Subjects with active head and neck malignant tumours.
9. Pregnant and lactating women.
10. Subjects who underwent treatment for headache or bruxism in last 6 months prior to their enrollment in RCT study
11. Studies utilised homeopathic therapy as a comparative therapy.
12. Narrative and systematic reviews, case reports, in vitro studies, in vivo animal studies, commentaries, interviews, updates or case series.
13. A necessity of initiating the use of any type of medications during any of phase of the study.
14. Studies investigating acute TMD or acute versus (vs.) chronic TMD.

### 2.7. Types of Outcomes Measures

#### 2.7.1. Primary Outcomes

Pain intensity reduction from baseline up to the end of follow-up utilising qualitative (patient-reported outcomes; subjective) and quantitative measures (objective) (Table 1).

#### 2.7.2. Secondary Outcomes

1. Functional improvement (muscles movements: mouth opening and closing and chewing) from baseline up to the end of follow-up.
2. Reduction in anxiety/depression and improved QoL from baseline up to the end of follow-up.

### 2.8. Data Extraction

A careful selection of the eligible studies from the search engines was carried out by two reviewers independently (R.H. and S.D.). They performed the review, assessment and data extraction for each eligible study. Each study received an identification with the name of the first author, year of publication and origin. A tabular representation of additional relevant information, such as the impact factor of the journal, study design, sample size, participants’ demographical data, baseline characteristics, intervention and comparator groups, type of light sources (laser or LEDs), number and location of trigger points (TP), utilised laser parameters, treatment frequency, follow-up duration, methods of outcome evaluation, statistical tests performed, results and conclusions, were harvested from each eligible study.

### 2.9. Qualitive Analysis 

Two review authors (R.H. and S.D.) independently assessed the risk of bias for each RCT study included in this review, using the criteria described in the revised Cochrane Risk-of-Bias (RoB) tool for randomised trials, version 2.0 (RoB 2) [42,43,44]. The performed assessment of risk of bias was based on the following domains:

1. Bias arising from the randomisation process.
2. Bias due to deviations from intended interventions.
3. Bias due to missing outcome data.
4. Bias in measurement of the outcome.
5. Bias in selection of the reported result.

Contingent on fulfilment of the above criteria, the chosen studies were governed as low, moderate or high RoB. Any incongruities between the two review authors’ assessments were settled via discussion with a third author (S.B.), as well as the “discrepancy check” feature in RoB 2, which was used to obtain consensual answers for the quality assessment of included studies.

### 2.10. Statistical Analysis of Data

A meta-analysis of the data of interest extracted from the eligible studies was performed, using RevMan (Version 5.4.1) [45]. A random effects meta-analysis for continuous outcomes was conducted to assess the heterogeneity amongst the included studies. Relevant data on the primary outcome VAS and secondary outcomes, maximum mouth opening (MMO) and PPT, was extracted from the included studies. Data were collected from the baseline evaluation up to the final follow-up evaluation of the eligible studies. Treatment effects were calculated through pooled standardised mean differences (SMDs) with associated 95% confidence intervals (95% CIs) and pooled overall effect was considered statistically significant when *p* < 0.05 [46]. Forest plots were visually inspected to identify statistical heterogeneity through the presence of outlier studies [46]. Additionally, *I*^2^ statistics for homogeneity that ranged from 0–100% with the following interpretation: 0% = no evidence of heterogeneity; 30–60% = moderate heterogeneity; and 75–100% = high heterogeneity, were calculated [47]. In order to evaluate the results after negation of heterogenous studies, a sensitivity analysis was performed [48]. The presence of publication bias was analysed by visual assessment of funnel plot symmetry [49].

## 3. Results

### 3.1. Study Selection

**Figure 2:**
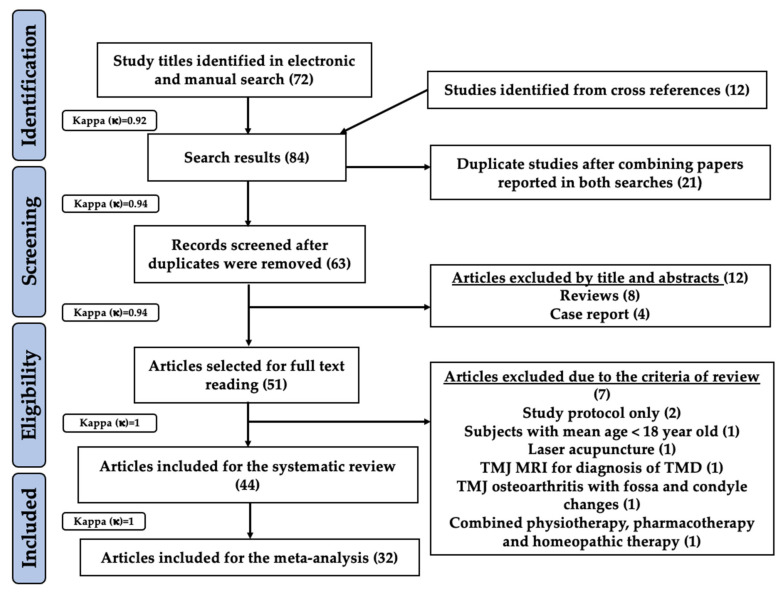
PRISMA flow-chart of selected criteria for the included articles [37].

A combined electronic and manual search revealed 72 study titles which were possibly eligible for this systematic review and meta-analysis. Twelve study titles were obtained from cross-references resulting in a total of 84 eligible study titles in the preliminary screening (inter-reviewer agreement, κ = 0.92). After evaluation for duplication, 21 articles were excluded and the remaining 63 records underwent further evaluation (inter-reviewer agreement, κ = 0.94). Based on their titles and abstracts, 12 articles were excluded mainly due to inappropriate study design (inter-reviewer agreement, κ = 0.94). Thus, 51 articles were assessed based on eligibility criteria. Seven studies were excluded due to the following reasons: two studies presented with a study protocol only [50,51], and one study each was excluded for the following reasons: subjects with mean age below 18 year old [52]; laser acupuncture [53]; TMJ magnetic resonance imaging (MRI) for diagnosis of TMD [54]; TMJ osteoarthritis with fossa ad condyle changes [55]; and utilisation of combined physiotherapeutic, pharmacotherapy and homeopathic measures [56] (inter-reviewer agreement, κ = 1). Consequently, 44 out of 51 full text articles were included and analysed in the present systematic review [57,58,59,60,61,62,63,64,65,66,67,68,69,70,71,72,73,74,75,76,77,78,79,80,81,82,83,84,85,86,87,88,89,90,91,92,93,94,95,96,97,98,99,100]. Furthermore, 32 out of 44 studies qualified for a meta-analysis [57,58,60,61,62,63,64,65,66,67,68,69,70,71,72,73,74,77,78,81,83,84,85,86,88,89,91,95,96,97,99,100] (inter-reviewer agreement, κ = 1). The PRISMA flow diagram for search strategy utilised in the present systematic review and meta-analysis is illustrated in Figure 2.

### 3.2. Characteristics of the Study Populations (Table S1)

#### 3.2.1. Sample Size

The sample size (n) distribution amongst the included studies was as follows; n > 50 in 10 studies [58,64,65,69,82,89,90,91,93,98], n = 40–50 in 15 studies [59,61,62,67,68,70,73,83,84,85,87,88,92,95,99], n = 30–40 in 7 studies [72,73,75,78,81,95,98], n = 20–30 in 7 studies [57,58,76,77,80,87,97] and n = 10–20 in 5 studies [61,64,67,79,82].

#### 3.2.2. Racial Background

For the purpose of this systematic review and meta-analysis, relevant reported data on patients’ racial background in the included studies was sought as follows: Black, Black/Caucasian, non-Caucasian. All the included studies failed to provide information regarding the phenotypic characteristics of the recruited subjects, apart from one study [88], which reported their subjects’ racial backgrounds under the category of Black/Caucasian (n = 44, 42 “White” patients, 2 “Black” patients).

#### 3.2.3. Gender Distribution

Amongst the included studies, gender distribution was noted as follows: more than a 50% female population in 27 studies [57,58,59,62,65,67,68,69,70,71,73,74,75,78,79,80,81,82,83,84,85,86,87,88,89,91,97,98,100], an only-female population in eight studies [61,72,76,77,90,93,95,99] and an approximately equal proportion of both males and female population in three studies [63,92,96]. Additionally, five studies failed to report any relevant information on gender distribution in their respective studies [60,64,66,78,84].

#### 3.2.4. Age Distribution

Five out of 44 studies included patients within the cohort of less than 18 years old and above 70 years old [1,2,3,11,14], one study included patients between 13 and 63 years old [1] and the other ranged between 15 and 55 years [14], whereas three studies included patients in an age range of 16 to 70 years old [2,3,11]. Twelve studies were conducted on patients with a mean age in the range of 25 to 35 years [13,17,29,30,32,34,35,36,37,39,42,44]. Eleven studies were conducted on patients with a mean age ranging from 35 to 45 years [6,9,12,15,18,21,24,25,26,27,40], two studies had patient age groups in the range of 18 to 25 years [38,41] and one study included patients more than 45 years old [33]. Additionally, 13 studies failed to provide a mean age in their respective studies [4,5,7,8,10,16,19,20,22,23,28,31,43] with a variation in the range of 18 to 60 years.

#### 3.2.5. Presented Symptoms

The majority of the included studies reported a combination of one or more of the following symptoms upon their first visit to the hospital; myofascial pain in 13 studies [59,67,68,72,76,78,80,81,82,94,97,98,100], orofacial pain in nine studies [58,69,71,73,74,75,87,96,99], MPDS in nine studies [66,67,78,81,96,97,98,99,100], limited MM in five studies [58,66,75,87,91], disc displacement in five studies [57,60,64,70,97], whereas capsulitis/synovitis [57,60,64] and limited mouth opening [77,84,89] were in three studies each. Two studies presented with TMJ clicking [87,90], tooth wear [61,91] and muscle disorders [52,77]. However, eleven studies did not specify the origin of the pain [60,61,62,63,64,66,79,85,86,87,88]. One study each reported patients with TMJ arthralgia [59], joint cracking [61], chewing difficulties [58], joint rigidity [61], head and neck pain [84] and joint noises [85]. 

With regards to the duration of presented pain symptoms prior to treatment, seven studies reported chronic pain over six months [57,62,65,69,71,74,90], whereas two studies reported pain for three to six months [93,96]. 

#### 3.2.6. Aetiology of TMD

The main TMD aetiology of recruited subjects in 39 out of 44 studies was related to myofascial pain disorder (extra-articular origin) [58,59,61,62,63,65,66,67,68,69,71,96,98,99,100], whereas in the remaining five studies [57,60,64,70,97], the aetiology was of mixed intra and extra-articular origins, distributed as follows: an inflammatory origin (capsulitis and synovitis) in three studies [57,60,64], osteoarthritis in one study [70] and disc displacement disorder with reduction in five studies [57,60,64,70,97].

#### 3.2.7. Affected Area

The affected areas in the included studies were categorized as follows: TMJ in nine studies [57,59,60,63,65,70,88,96,99]; extraoral (EO) masticatory muscles in eight studies [66,68,76,78,80,81,83,97]; intraoral (IO) masticatory muscles in one study [95]; TMJ and masticatory muscles (EO) in 10 studies [69,73,74,75,85,90,91,92,93,98]; TMJ and masticatory muscles (EO and IO) in seven studies [11,16,21,28,33,38,44]; TMJ, masticatory muscles (EO) and cervical (neck muscles) in four studies [61,62,71,87]; TMJ, masticatory (EO and IO) and cervical muscles in four studies [58,64,79,82]; and masticatory (EO and IO) and cervical muscles in one study [86].

In terms of allocation of symptoms/affected area at baseline, four out of 44 studies specified unilateral TMJ symptom presentation [65,67,72,92], whereas three studies specified bilateral TMJ involvement [58,76,77]. Interestingly, three studies reported inclusion of patients with either unilateral or bilateral TMJ involvement [57,88,100], which were as follows; in one study, five patients in each group (two groups with 15 patients in each, n = 30) presented with unilateral TMJ involvement, and the remaining 20 patients showed bilateral TMJ involvement [57]; in one study, seven patients (n = 44) presented with unilateral TMJ involvement and the rest of the patients showed bilateral TMJ involvement [88]; and in one study, 16 patients (n = 44) presented with unilateral TMJ involvement and the rest of the patients showed bilateral TMJ involvement [100].

#### 3.2.8. Functionality Problems

Pain was considered as the most commonly reported functionality problem in the majority of the included studies [57,58,59,60,61,62,63,64,65,66,67,68,69,71,72,73,74,75,76,78,79,80,81,82,84,85,86,87,88,89,90,92,93,94,95,96,97,98,99,100]. Five studies reported limited MM [58,66,75,84,87,89,91,98], whereas the other commonly associated functionality problems reported in the included studies were TMJ clicking [87,91], joint cracking [61], chewing difficulties [58], joint rigidity [61], muscle tenderness [64] and joint noises [85]. One study failed to report the relevant information on functionality problems [70]. 

### 3.3. Study Characteristics

#### 3.3.1. Country of Origin

Out of 44 studies, 23 were conducted in Brazil [57,60,61,62,64,66,68,73,74,75,76,79,80,82,83,88,89,90,93,95,97,98,100], five studies each in Iran [63,67,77,84,91] and Turkey [58,69,71,78,81], three studies in India [85,96,99] and one study each in the following countries: Czech Republic [59], Italy [70], Austria [65], UK [72] U.A.E. [87], Saudi Arabia [92] and Iraq [94] (Table S1).

#### 3.3.2. Study Design 

All the included studies were conducted using a parallel study design (Table S1).

#### 3.3.3. Intervention Group

Several inconsistencies were observed amongst the included studies [57,58,59,60,61,62,63,64,65,66,67,68,69,70,71,72,73,74,75,76,77,78,79,80,81,82,83,84,85,86,87,88,89,90,91,92,93,94,95,96,97,98,99,100], in terms of the intervention groups, which are outlined in Table S1. 

Out of 44 studies, 20 evaluated the effect of PBMT compared to a placebo (sham PBMT) group [57,58,60,61,62,63,64,65,71,77,80,81,83,85,87,90,92,93,94,100]. Two studies assessed different PBM therapeutic doses [59,88]. In terms of different treatment strategies for TMD, two studies each evaluated the role of PBMT vs. occlusal splints [69,97] and TENS [86,99], respectively. One study compared PBMT vs. drug therapy [91], manual therapy (MT) [89] and ultrasound therapy [96], respectively. 

A total of 16 studies employed more than two intervention groups [66,67,68,70,72,73,74,75,76,79,82,84,89,95,98]. The distribution of these studies with multiple intervention groups was as follows: four studies compared PBMT at different doses to sham PBMT [66,68,72,84], two studies each compared PBMT vs. occlusal splints and sham PBMT [78,98] and compared PBMT at different wavelengths and with sham PBMT [67,95], respectively, whereas one study compared PBMT vs. drug and sham PBMT [70]. One study compared PBMT with drug therapy, as well as a combination of PBMT and drug therapy [73] and MT, as well as a combination of PBMT and MT [89], respectively. One study each compared PBMT vs. needle therapy and sham PBMT [76] and PBMT vs. TENS and sham PBMT [84]. Three studies compared PBMT at different wavelengths and at different doses [75,79,82].

#### 3.3.4. Documentation of Reported PBM Irradiation Parameters

Table S2 describes the utilised PBM-laser/LEDs dosimetry in the eligible studies. These parameters are listed below:

##### Utilised Wavelength

In terms of laser PBMT, the majority of studies were performed utilising a variety of diode laser wavelengths; 19 studies utilised wavelengths ranging from 800–890 nm [58,59,62,69,70,71,73,77,80,81,83,84,85,88,89,91,97,98], nine studies utilised 750–790 nm [57,60,64,66,68,74,76,90,93], four studies with 600–680 nm wavelengths [65,96,99,100] and five studies utilised 900–980 nm wavelengths [61,63,86,87,92], whereas one study utilised 1064 nm [78]. Three studies [67,79,82] used multiple laser PBM wavelengths for different interventions groups, as follows: 660 and 890 [67], 660 and 795 [79] and 660 and 790 nm [82]. 

In terms of LED PBMT, one study utilised 660 nm [94]. Two studies [75,95] utilised LEDs (red and infrared) combined with infrared laser, as follows: 630 ± 10 and 850 ± 10 nm red LEDs and 780 nm infrared laser [75], whereas the other study utilised a 905 nm laser diode combined with LEDs of eight clustered heads, of which four were 640 nm and the other four were of 875 nm infrared [95].

##### Emission Mode

Emission mode was reported in 26 studies. The following are the utilised emission modes: a continuous wave emission mode (CW) for laser PBMT in 22 studies [60,61,64,65,66,68,69,71,74,80,81,84,85,86,87,88,89,90,92,93,97,100], pulsed mode for laser PBMT in three studies [63,77,78] and super-pulsed mode only in one study [70]. 

A combination of CW and pulsed emission modes for multiple laser wavelengths was utilised in one study [67]. A combination of pulsed and super-pulsed modes for combined laser-LEDs-induced PBM was employed in one study [95]. A total of 16 studies did not report the emission mode [57,58,59,62,72,73,75,76,79,82,83,86,91,94,96,99]. 

##### Pulse Width (s, μs) and Frequency (Hz)

The frequency for laser-PBMT in pulsed mode was mentioned in three studies [63,67,77] in the range between 80 and 1500 Hz. The frequency for laser PBMT in super-pulsed mode was mentioned in one study [70] and ranged between 1 and 50 kHz. The frequency values for a combined super-pulsed laser and pulsed LED PBM that were found in one study [95] were 1000 and 2–16 Hz, respectively. Only two studies [70,77] reported a pulse width of <200 nanoseconds (ns) and 1 microsecond (μs), respectively.

##### Laser/LED Tip-Tissue Distance (Contact/Non-Contact)

A total of 35 studies provided information on the laser tip-tissue contact, out of which 29 studies utilised a contact mode of application [58,59,60,61,62,63,64,65,66,67,68,70,71,72,74,75,76,77,88,89,90,91,93,95,96,97,98,99,100] and six studies utilised non-contact mode where tip-to-tissue distance ranged between 2 mm and 3 cm [69,78,81,85,87,92].

##### Reported Energy (J)

A total of 19 studies reported energy parameters [62,63,67,72,73,75,77,80,83,88,89,91,92,94,95,96,97,99,100]. Their data distribution was as follows: 2–4 J/point in three studies [62,91,99] and 6–10 J/point in three studies [67,77,97], whereas only one study reported 2.2 J/min [96]; cumulative energy reported in three studies ranged from 1.5 J in two studies [80,94] to 300 J in the third study [36]; total energy ranged from 6 to 128 J in four studies [63,73,83,100]; and multiple energy values for different points/sites ranged from 4 to 110 J/point in five studies [72,75,88,89,95]. 

##### Power Output and Therapeutic Power Output (W, mW)

The majority of the studies reported a power output in the range of 15 to 500 mW/point [57,59,60,61,62,64,65,66,67,68,69,70,71,72,73,74,75,76,77,78,80,81,82,83,84,85,86,87,88,89,90,91,92,93,94,95,96,97,98,99,100]. A power meter to measure the therapeutic power output, reaching the target tissues, was utilised in 11 out of 44 studies [61,65,67,70,73,75,77,83,88,89,95]. 

##### Energy Density (Dose or Fluence, J/cm^2^)

Energy density (fluence) was calculated in 38 studies [57,58,59,60,61,62,64,65,66,67,68,69,71,72,73,74,75,76,77,78,79,80,81,82,83,85,86,88,89,90,91,92,93,95,97,98,100] and its value was between 0.1 and 100 J/cm^2^/point.

##### Exposure Time (s)

A total of 39 studies reported the exposure time for irradiation and the values ranged from 10 to 120 s/point [57,58,60,61,62,63,64,65,67,68,69,70,71,73,74,75,77,78,80,81,82,83,84,85,86,87,88,89,90,91,92,93,94,95,96,97,98,99,100]. 

##### Frequency of the Treatment (Number of Sessions)

A total of 13 studies reported eight sessions [60,61,64,66,68,75,80,85,86,87,90,91,93], out of which 11 reported two sessions/week [60,61,64,66,68,75,80,87,90,91,93]. Their data distribution was as follows: two to three sessions/week in one study [85]; one study failed to specify [86]; and 10 sessions in eight studies [58,59,69,70,71,74,78,88]: two sessions/week [69,74], five sessions/week [70], three sessions/week [71,88], one session daily [78], one session daily except weekends [58] and interval not mentioned [59]. 

Six studies reported 4 to 4.5 sessions [62,73,76,84,94,100]. Their data distribution was as follows: 4.5 sessions/week [84], four sessions (72 hr interval between sessions one and two; 48 hr interval between sessions two and three; and 72 hr interval between sessions three and four) [76] and two sessions/week [73], respectively; and one session/week [62,94,100]. 

Four studies reported a total of 12 treatment sessions [41,77,81,89] distributed as follows: three sessions/week [77,81,89] and two sessions/week [97]. Four studies reported a total of six sessions [57,67,95,99], as follows: two sessions/week [57,67,99] and one study failed to mention the interval [95]. Whereas three studies reported three sessions (alternate day) [72,79,82] and two sessions (alternate days) in two studies [63,98]. 

One study each reported 20 sessions (two to three sessions/week) [65], 15 sessions (every alternate day) [96], five sessions (every alternate day) [92] and one session [83], respectively.

##### Duration of the Treatment

The total duration of the PBMT was reported in all included studies with a range of four days to eight weeks. 

##### Spot Size/Spot Area/Beam Diameter/Fibre-Tip Diameter Parameters

These parameters were addressed in the included studies, as follows; spot size in the range of 0.02 to 2.8 cm^2^ in nine studies [32,33,35,36,67,73,77,82,83,84,100] and 5 mm in one study [84], spot area of 0.028 cm^2^ in one study [80], beam diameter in six studies [69,71,74,81,97,100] in the range of 3.5 to 8 cm, beam area in three studies [61,67,97] in the range of 0.09 to 1 cm^2^ and tip diameter 300 μ in one study [85]. Additionally, two studies [59,95] mentioned the probe aperture size, which ranged from 4 to 0.2 cm^2^. An illuminated area of 0.5 cm^2^ was mentioned in one study [75], while the irradiated area was mentioned in two studies [97,100] in the range of 0.5 to 0.7 cm^2^/point. 

##### Methods of PBM Applications, and Number and Allocation of TP

a. Method of PBMT application

In total, 40 studies utilised an extraoral (EO) approach [57,58,59,60,61,62,63,64,65,66,68,69,70,71,72,73,74,75,76,77,78,80,81,82,83,85,86,87,88,89,90,91,92,93,94,96,97,98,99,100], one study utilised an intraoral (IO) approach [95] and two studies utilised a combined IO and EO approach [67,79]. One study failed to specify the relevant information [84]. 

The allocations of PBM irradiation were as follows: IO pterygoid muscles in one study (no. of TP and allocations unspecified) [95]; EO and IO of masseter, MPM and LPM (no. of TP unspecified) in one study [67]; whereas one study [79] utilised an EO approach for a region of TMJ (posterior ligament and lateral pole), EO masticatory muscles (temporalis, masseter, posterior mandibular and submandibular regions), cervical muscles (suboccipital, SCM, and trapezius) and IO muscles (LPM and temporal tendon) and masticatory muscles (no. of TP unspecified). Out of 44 studies, 21 reported a bilateral PBMT application protocol [57,58,60,61,64,70,73,74,75,76,77,80,81,83,86,88,89,90,95,97,100] and two studies specified unilateral PBMT application [72,92], whereas the remaining 21 studies did not specify.

b. Number of trigger points (TP)

Out of 44 studies, 24 specified TP, in which 23 studies mentioned the number of TP and their allocations in detail [57,58,60,61,64,68,69,71,72,73,74,75,80,81,83,88,89,90,92,97,98,99,100], whereas one study failed to specify the allocation of the TP but noted their numbers [91]. The remaining 20 studies failed to provide any of the above information [59,62,63,65,66,67,70,76,77,78,79,82,84,85,86,87,93,94,95,96].

c. Allocation of TP

The following 14 studies reported PBM applications on five or more TP/side: eight studies reported five points/side [61,64,71,75,80,83,90,92], two studies reported eight points/side [74,97], two studies reported six points/side [72,81], 12 points/side were reported in one study [89] and 10 points/side in one study [73]. However, one study reported 24 TP without specifying if they were the total TP or per side [98]. The following nine studies reported PBM applications on <5 points/side: five studies reported four points/side [58,68,88,99,100], three studies reported three points/side [57,69,91], one study utilised one point/side [60] 

The distribution of the TP allocation for the temporalis muscle noted in 12 studies [68,69,73,74,75,81,83,89,90,92,97] was categorised as follows: anterior, middle and posterior (three points/side) in five studies [73,81,83,89,90], anterior temporalis (one point/side) in five studies [68,69,71,74,97] and centre of temporalis muscle (one point/side) reported in one study [92]; however, one study did not specify the allocation in the temporalis muscle [75]. 

The distribution of the TP allocations for the masseter was as follows; superior, middle and inferior (three points/side) in eight studies [68,69,71,74,81,89,90,97], origin and insertion (2 points/side) [73], superior and inferior (two points/side) in one study [83], origin of masseter (1 point/side) in two studies [75,92]. 

Around the TMJ region, the number of the TP were between three and five points/side in three studies [75,89,90], of which in one study [89] the five-TP distribution was as follows: superior, anterior, lateral, posterior and postero-inferior to the condyle, whereas the distributions of five points/side of the other study [90] were related to the lateral pole of the mandible head as follows: lateral, superior, anterior, posterior and inferior regions. One study did not provide the exact allocation of three TP on TMJ [75]. 

In terms of the TMJ itself, the distribution of TP was as follows: the joint capsule (five points (lateral, posterior, superior, anterior, inferior)/side) in one study [73], the condylar region (five points (anterior, superior, posterior and posteroinferior points of the condylar position and in the external auditory meatus)/side) in one study [74] and pre-auricular region and external auditory meatus in four studies, of which two studies mentioned the application of one point inside the external auditory duct towards the retrodiscal region [60,61], one study applied four points/side (TP and allocation unspecified) [88] and one study failed to provide any of the above relevant information [59]. Furthermore, one study applied one point/side on superior SCM [71] while one study applied irradiation on SCM without specifying the exact allocation or number of TP [79].

In the line of the extrapolated data of laser documentations, it is noteworthy that five studies [57,60,64,70,97] recruited subjects with mixed TMD aetiology (Section 3.2.6), and did not apply different laser protocols for each category. Additionally, they did not identify the number of subjects according to aetiology category. 

#### 3.3.5. Follow-Up Assessment

The follow-up assessment ranged from 10 days up to one year amongst the included studies. The majority of the included studies performed re-evaluation at multiple follow-up visits. A short-term follow-up assessment of less than one month from baseline visit was performed in 11 out of 44 studies [59,67,71,72,76,78,83,88,92,95,99]. A total of 28 studies reported follow-up assessment ranging from one to three months [57,58,60,61,62,64,65,66,68,69,70,73,74,75,77,80,81,84,85,87,89,90,91,94,96,97,98,100], three studies from three to six months [80,82,86] and two studies reported follow-up assessment ranging from six months to one year [63,93] (Table S1).

#### 3.3.6. Assessment Methods 

Table 1 illustrates the quantitative and qualitive methods of assessment of pain intensity, functional problems, anxiety/depression and QoL, which were utilised in the eligible studies.

### 3.4. Qualitative Assessment

**Figure 3:**
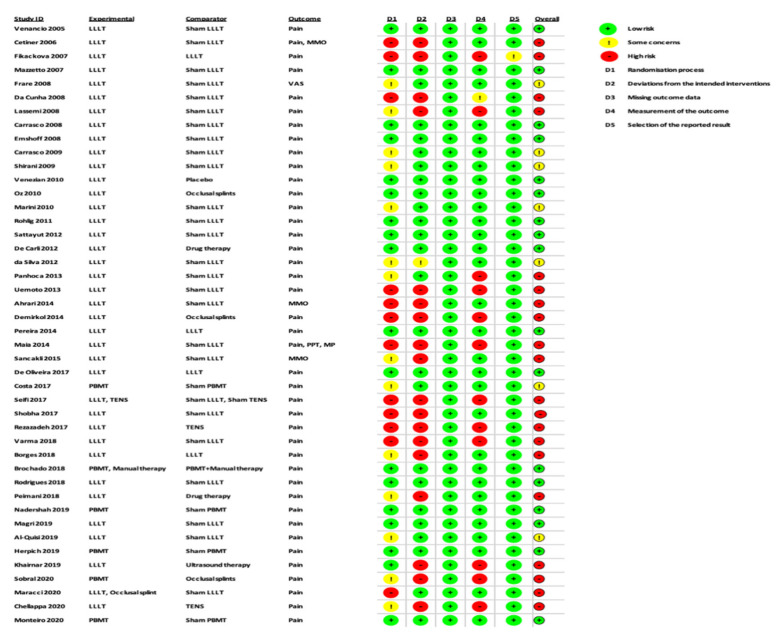
Risk of Bias assessment summary of the included studies based on the consensual answers of two individual assessors (R.H. and S.D.)

**Figure 4:**
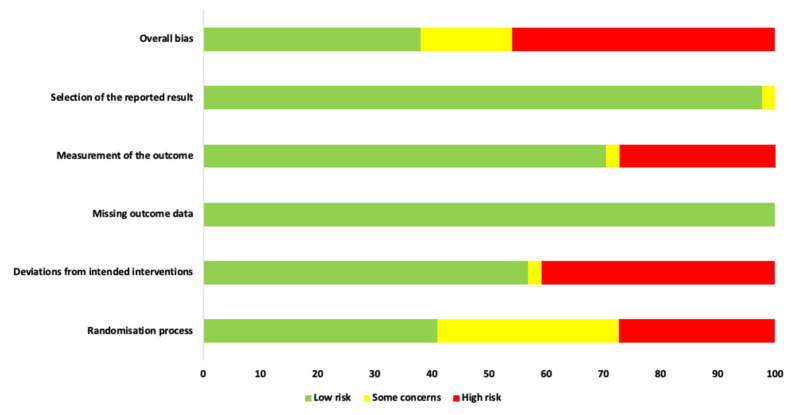
Risk of Bias assessment graph of the included studies expressed as percentages based on the consensual answers of two individual assessors (R.H. and S.D.).

All included studies were assessed for their quality, using the RoB 2 tool designed for in vivo human RCTs, as shown in Figure 3 and Figure 4. This tool is the latest version, which was used to execute a qualitative assessment [34,35,36,37,38,39,40,41]. Figure 3 is an illustration of the RoB assessment summary of all the eligible studies, whereas Figure 4 is a domain-wise graphical representation of percentage RoB score evaluated using this tool. Both figures represent the consensual answers verified using the “discrepancy check” feature of the RoB 2 tool, across two independent reviewers (R.H. and S.D.) (inter-reviewer agreement, κ = 0.92). Of the included trials, 40% were at low risk of inadequate randomization, whereas 32 and 28% of the included trials had some concerns or were at high risk, respectively. Of the included studies, 59% were at low risk of deviations from intended interventions, whereas 39 and 2% were at high risk or had some concerns, respectively.

All included papers were at a low risk (100%) in terms of substantial evidence for reporting missing outcome data. In terms of reporting outcome measurement, 70% of the included trials were at low risk, 28% were at high risk and 2% had some concerns. Of the included studies, 98% were at low risk of bias for selective reporting of the results and 2% of the studies had some concerns. Overall, 38% (17 out of 44 studies) reported a low risk of bias [57,60,64,65,68,69,71,72,73,79,82,89,90,92,93,95,100], while 46% (20 out of 44 studies) were at high risk [58,59,62,63,75,76,77,78,80,81,83,84,85,86,87,88,91,96,97,98,99] and 16% (7 out of 44 studies) had some concerns [61,66,67,70,74,83,94]. 

### 3.5. Impact Factor of the Published Papers

Ten out of 44 studies were published in high-impact-factor (IF) journals of “>2” [67,70,73,75,77,78,80,88,94,95]. A total of 16 studies were published in moderate-IF “between 1–2” journals [58,59,65,66,68,74,76,79,81,82,83,89,90,93,98,100]. Seven studies were published in low-IF “<1” journals [57,60,61,62,64,69,71]. The journal impact factors for 11 studies were unavailable, which might imply a low impact factor [63,72,84,85,86,87,91,92,96,97,99].

### 3.6. Quantitative Assessment

#### 3.6.1. Outcome Variables

The treatment outcomes were broadly based on three categories; pain reduction, functionality improvement and anxiety reduction/QoL improvement (Table S3). These outcomes were assessed with qualitative and quantitative measures.

In terms of pain reduction assessment, qualitative measurements (VAS, SSI, OMES, etc.) were utilised in 42 out of 44 studies [57,58,59,60,61,62,63,64,65,66,67,68,69,70,71,72,73,74,75,76,77,78,79,80,81,83,84,85,86,87,88,89,90,91,92,93,94,95,96,97,99,100] and quantitative measurements (PPT, Kaplan–Meier method, etc.) were used in 9 studies [64,69,71,72,76,78,80,81,83]. A total of 33 out of 42 studies [58,59,60,61,63,64,66,67,68,70,71,72,74,76,79,80,81,83,84,86,87,88,89,90,91,92,93,94,95,96,97] and four out of nine studies showed a statistically significant improvement in qualitative and quantitative measurements of pain reduction, respectively [71,72,80,81] (Table S3). 

The functionality improvement assessments were based on the qualitative measurements (functional scale, etc.) utilised in one study [95], and quantitative measurements (CMI, EMG, AROM, jaw kinesiology, etc.) that were used in 34 studies [57,58,62,63,64,68,69,70,71,72,73,74,75,76,77,78,80,81,82,84,85,86,88,89,91,93,95,96,97,98,99,100]. Statistically significant results were noted in the study which qualitatively measured functionality improvement [95], whereas 18 out of 34 studies showed statistically significant quantitative functionality improvement [58,63,68,70,71,72,74,80,81,84,86,88,89,91,93,96,97,100] (Table S3). 

Anxiety reduction/QoL improvement was qualitatively measured, utilising EuroQoL-5D, BAI, OHIP-14, etc. (Table 1) in four out of 44 studies [79,89,97,98], out of which three studies showed statistically significant improvement [79,89,97] (Table S3).

Out of 44 eligible studies, 33 with relevant numerical data for the primary outcome measure (pain reduction assessment by qualitative measurement with VAS) contributed to this meta-analysis [57,58,60,61,62,63,64,65,66,67,68,69,70,71,73,74,78,81,83,84,85,86,88,89,91,95,96,97,99,100]. 

**Figure 5:**
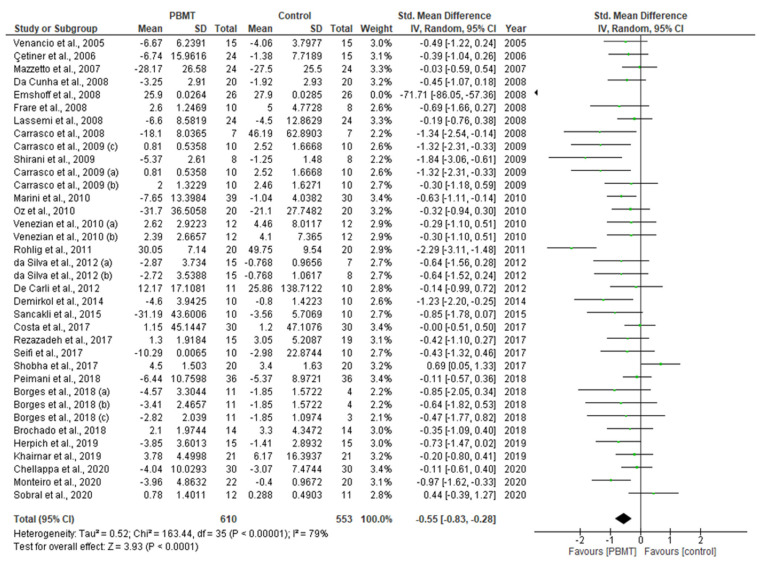
Forest plot for primary outcome pain assessment (VAS score) from baseline up to the final follow-up timepoint.

Data extracted from a total of 1163 patients, evaluated from baseline up to the final follow-up evaluation for each study, was pooled to reveal a statistically significant intergroup difference (SMD = −0.55; 95% CI = −0.82 to −0.27; Z = 3.90 (*p* < 0.001)), favouring the PBMT group, along with a borderline high heterogeneity (T^2^ = 0.51; χ^2^ = 161.97 (*p* < 0.0001); I^2^ = 78%) amongst the included studies (Figure 5). 

**Figure 6:**
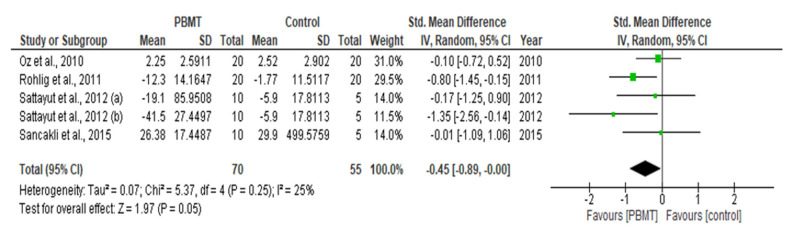
Forest plot for secondary outcome quantitative pain reduction assessment (PPT) from baseline up to the final follow-up timepoint.

**Figure 7:**
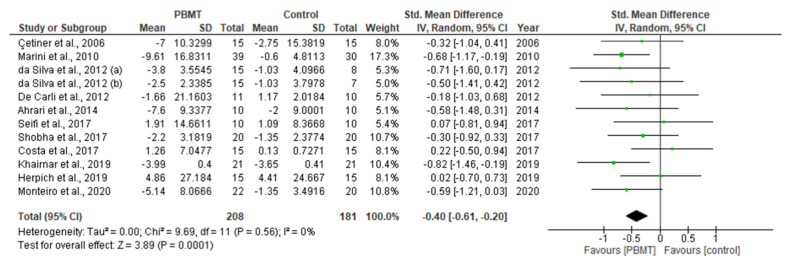
Forest plot for secondary outcome functionality improvement (MMO) from baseline up to the final follow-up timepoint.

Assessment of numerical data extracted from four studies comprising 125 patients on PPT, which is a quantitative measurement of pain reduction, revealed a statistically significant intergroup difference (SMD = −0.45; 95% CI = −0.89 to 0.00; Z = 1.97 (*p* = 0.05)) favouring the PBMT group, along with low heterogeneity (T^2^ = 0.07; χ^2^ = 5.37 (*p* = 0.25); I^2^ = 25%) amongst the included studies (Figure 6) [69,71,72,81]. Furthermore, an assessment of numerical data extracted from 11 studies comprising 389 patients on MMO, which is a quantitative measurement of functionality improvement, revealed a statistically significant intergroup difference favouring the PBMT group (SMD = −0.40; 95% CI = −0.61 to −0.20; Z = 3.89 (*p* = 0.0001)) with no evidence of heterogeneity (T^2^ = 0.00; χ^2^ = 9.69 (*p* = 0.56); I^2^ = 0%) amongst the included studies (Figure 7) [58,70,73,74,77,83,84,85,95,96,100].

A meta-analysis on other secondary outcomes could not be conducted owing to the disparity in scoring methodology and incomplete or incomparable numerical data.

#### 3.6.2. Subgroup Analysis

**Figure 8:**
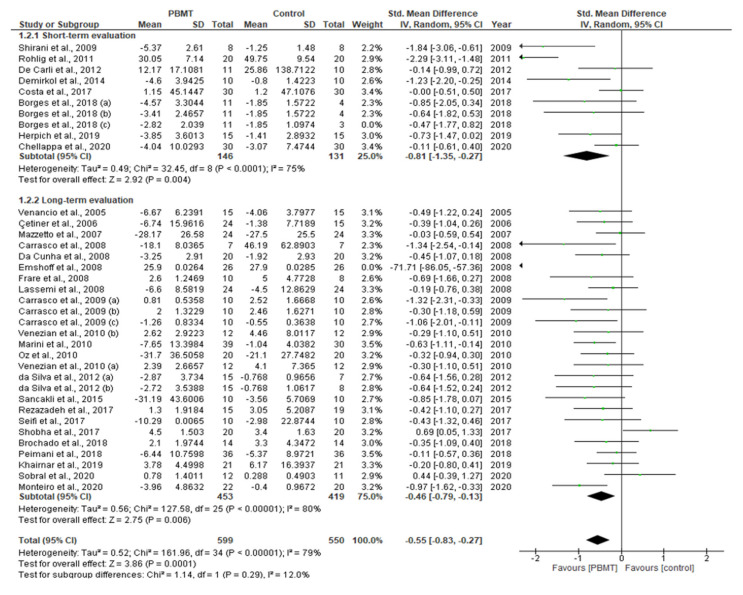
Forest plot with sub-group analysis for primary outcome qualitative pain reduction assessment (VAS score) from baseline up to the final follow-up timepoint.

Owing to the high heterogeneity in the meta-analytical assessment of the primary outcome measure, pain reduction, a subgroup analysis on the role of length of follow-up evaluation was conducted. Numerical data on short-term evaluations of less than 30 days, obtained from eight out of 30 studies [67,71,73,78,83,88,95,99] accounting for 291 patients, revealed a statistically significant intergroup difference favouring the PBMT group (SMD = −0.78; 95% CI = −1.29 to −0.27; Z = 3.02 (*p* = 0.003)) with a high heterogeneity (T^2^ = 0.45; χ^2^ = 32.47 (*p* = 0.00002); I^2^ = 72%) amongst the included studies (Figure 8). Similarly, numerical data on long-term evaluations of 30 days and longer which were obtained from 22 out of 30 studies [57,58,60,61,62,63,64,65,66,68,69,70,74,81,84,85,86,89,91,96,97,100] revealed statistically significant intergroup differences (SMD = −0.46.18; 95% CI = −0.79 to −0.13; Z = 2.75 (*p* = 0.006)), favouring the PBMT group, with a high heterogeneity (Τ^2^ = 0.56; χ^2^ = 127.58 (*p* < 0.0001); I^2^ = 80%) amongst the included studies (Figure 8). 

A test of subgroup differences revealed very low heterogeneity amongst the short- and long-term evaluation protocols (χ^2^ = 1.06 (*p* = 0.30); I^2^ = 5.3%), which indicated that the length of follow-up evaluation was not a contributing factor towards the highly heterogenous results obtained in the abovementioned meta-analysis (Figure 8).

#### 3.6.3. Sensitivity Analysis 

**Figure 9:**
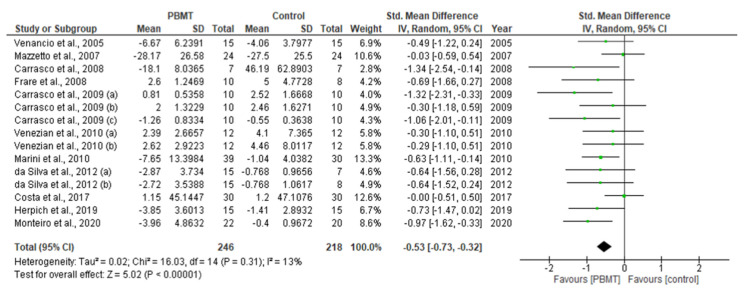
Sensitivity analysis for primary outcome qualitative pain reduction assessment (VAS score) from baseline up to the final follow-up timepoint.

A sensitivity analysis was conducted due to the noteworthy heterogeneity, arising from 16 outlier studies for qualitative pain reduction assessment through VAS analysis. These outlier studies had a low study quality and were detected as outliers upon visual inspection of Forest plots [58,62,63,65,67,71,78,81,84,85,86,88,91,96,97,99]. The remaining 14 studies were subjected to a sensitivity analysis which revealed a statistically significant intergroup difference (SMD = −0.53; 95% CI = −0.73 to −0.32; Z = 5.02 (*p* < 0.0001)) with low heterogeneity (Τ^2^ = 0.02; χ^2^ = 16.03 (*p* = 0.31); I^2^ = 13%) (Figure 9).

#### 3.6.4. Publication Bias

**Figure 10:**
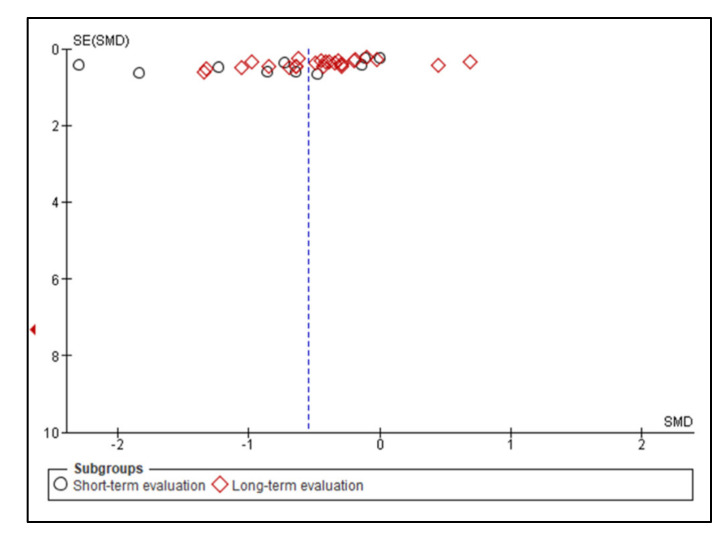
Funnel plot summary for primary outcome qualitative pain reduction assessment (PPT) from baseline up to the final follow-up timepoint.

**Figure 11:**
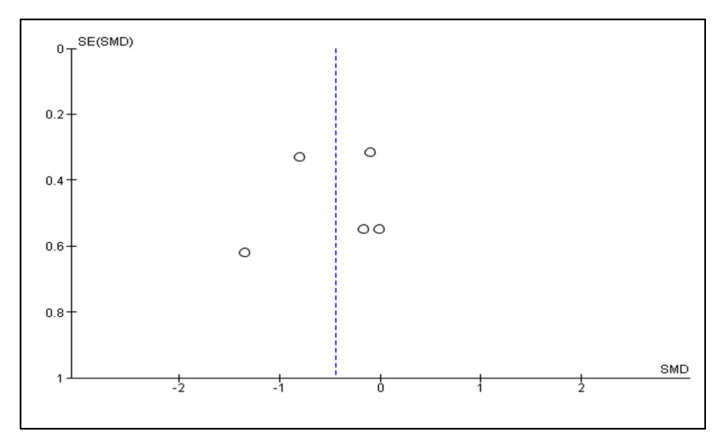
Funnel plot summary for secondary outcome quantitative pain reduction assessment (VAS score) from baseline up to the final follow-up timepoint.

**Figure 12:**
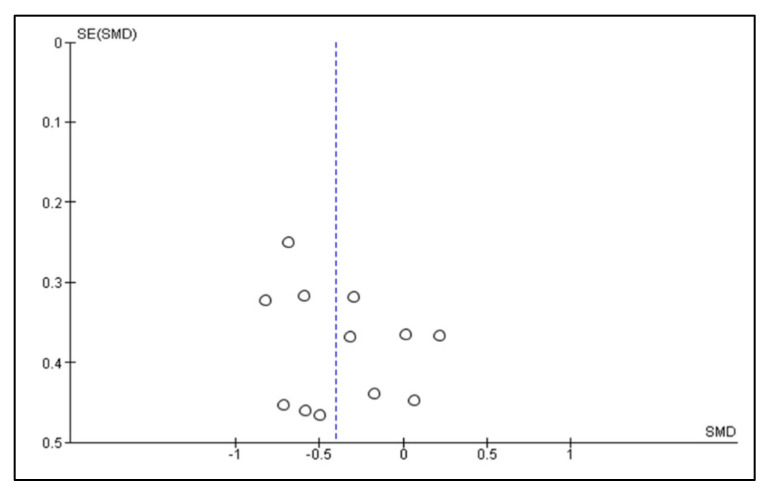
Funnel plot summary for secondary functionality improvement (MMO) from baseline up to the final follow-up timepoint.

Visual inspection of funnel plots for pain reduction assessment by VAS, PPT and functionality improvement assessment by MMO revealed only mild asymmetries, respectively, indicating a not significant risk of publication bias in this meta-analysis (Figure 10, Figure 11 and Figure 12).

## 4. Discussion

This systematic review and meta-analysis scrutinised RCTs that recruited subjects diagnosed with TMD, according to RDC/TMD [32,33], and treated them with PBMT of various wavelengths, as a single or dual treatment modality compared to the placebo or several conventional standards of care, or different laser PBM or LED PBM. Despite the inconsistencies and diversities in the reported PBM parameters, variable assessment tools, missing data and variable study designs, the majority of the included studies reported positive results in favour of the effectiveness of PBMT. Hence, this systematic review and meta-analysis, for the first time, has addressed the methodology and PBM protocols standardisation by proposing suggested recommendations, which can only be used to pave the roadmap for future extensive research in TMD management. In this context, our in-depth review has revealed the following important lacunae and the evidence-based science and practice to bridge them.

### 4.1. Characteristics of the Reported Recruited Subjects (Population’s Phenotype)

In terms of the gender of recruited subjects, the female gender was predominant (>50%) in 27 of the included study samples of the present systematic review [57,58,59,62,65,67,68,69,70,71,73,74,75,78,79,80,81,82,83,84,85,86,87,88,89,91,97,98,100], whereas only-female populations were used in eight studies [61,72,76,77,90,93,95,99]. This was supported by the findings of a review conducted by Bueno et al. (2018), which showed that subjects of the female gender were at double the risk of developing TMD than males [101]. Hence, it is not yet clear and well understood which aspect of female biology, psychology or social roles might predispose them to TMD. The differences between gender might be related to multifactorial elements, such as hormonal [102], cultural or social factors [103], stress tolerance [104], pain threshold and sensitivity and health-seeking behaviours [105,106]. Understanding the underlying triggers can determine the clinical approach to TMD treatment. Notably, in this review three studies recruited mixed-gender participants [63,92,96].

It is noteworthy that gender and age have a great influence on skin thickness [107,108,109]. In this context, only five out of 44 eligible studies included the following disproportional age range of their cohorts: 13–63 years old [1], 15–55 years old [14] and 16–70 years old [2,3,11]. 

In terms of racial background, only one study by Borges et al. (2018) reported the characteristics of the recruited subjects’ racial backgrounds under the category of Black/Caucasian (n = 44: 42 “White” and 2 “Black” patients) [88]. However, they failed to utilise different laser parameters and the number of subject allocations was uneven. The remaining 43 studies failed to report the racial background of their recruited subjects. It is noteworthy that skin colour plays an important role in the scattering and absorption of the photonic energy [110,111], which should be considered when a PBM (laser or LEDs) parameter protocol is formulated. Regrettably, this was not employed by Borges et al. (2018); hence, their results cannot be reproducible [88]. 

The above notes highlight the possibility of discrepancy and diversity in the included studies’ findings. Hence, standardisation of an equal number of recruited subjects of the same racial background, gender, suitable age range and muscle volume are the most fundamental key factors for recruited TMD population characteristics. Based on this, the PBM laser parameters can be formulated. 

### 4.2. Methodology Quality 

#### 4.2.1. Evaluation of Study Design

The investigators need to pre-determine the eligibility criteria for the population included in the trial. Interestingly, three RCT studies [57,88,100] included in their inclusive criteria TMD subjects with unilateral and bilateral TMJ symptoms; however, the numbers of recruited subjects were uneven. The distribution of the recruited subjects in relation to these parameters were as follows: 10/30 patients presented with unilateral and 20/30 bilateral [57], 7/44 patients unilateral TMJ and 37/44 bilateral [88] and 16/44 patients with unilateral and 28/44 bilateral [100]. This can have a great impact on the reported outcomes; however, the findings were positive. The majority of the high risk of bias in the quality of available evidence was derived from randomisation processes (28%, arising from 12 out of 44 studies) [58,59,62,76,77,78,80,84,85,86,87,98]. Hence, a robust randomisation process is required to ensure validation of the findings.

#### 4.2.2. Diagnostic Criteria

Within our review eligibility criteria, we included all the studies that utilized RDC/TMD tools to assess TMD symptoms. In this review, two studies [61,84] mentioned symptoms related to the neck; however, these were not addressed for PBM applications. Interestingly, two studies [71,79] did not report the neck symptoms of their participants, but the TP of the cervical muscles were irradiated. The remaining 40 studies neither reported neck symptoms nor if the TP of the cervical muscles were irradiated.

Based on the above notes, it is important to highlight the factors that contributed to the inconsistency of findings, as follows:

1. Despite RDC/TMD being the most common TMD diagnostic tool utilised by various researchers, there are three fundamental downsides to it, which are as follows [112]: (a) Relatively limited use due to the diversity of clinically presented TMD symptoms. Therefore, this tool can be utilised but many of the presented symptoms will not fit in one or any category. (b) They do not account for cervical spine involvement, which is crucial to thorough evaluation and management. (c) Various identified patients’ experiencing pain symptoms such as hyperalgesia and/or allodynia should be managed accordingly, despite the fact that these variables are not addressed by RDC/TMD criteria.
2. Regardless of if TMD participants have symptoms in the cervical regions, a full clinical assessment is required and the cervical muscles’ TP need to be addressed in the irradiation protocol. This is due to an altered neuro-biomechanical function of the cervical spine, which can apply stress on the TMJ, causing TMD. There is evidence to support that the craniomandibular region and upper cervical spine are related from anatomical, biochemical and neurophysiological standpoints [113,114], due to a neuroanatomical link between the orofacial and cervical regions, as well changes in the isometric strength of cervical flexors, according to the bite position of TMD patients [115,116]. Hence, the manual muscle test (MMT) is a reliable and useful clinical diagnostic tool for cervical muscles assessment to be considered [117].

Based on the above notes, a combination of RDC/TMD and MMT tools in TMD diagnosis and measurement of the presence and severity of symptoms could offer a standardised consensus among researchers and clinicians.

#### 4.2.3. Assessment of the Outcome Measures

In many previous studies of PBMT in TMD pain patients, the focus has been on jaw movements or self-reported pain intensity; however, a few studies have determined changes in patients’ somatosensory function using more objective testing methods [118,119]. Simple rating scales, such as NRS or VAS have been considered the most reliable tools to evaluate self-reported pain intensity in clinical practice; however, this approach may be an oversimplification of complex biopsychosocial pain problems and may possibly result in an underestimation or overestimation of reported pain [19]. All the included studies in this review utilised either of the above pain assessment tools. The present review’s authors recommend utilisation of the core outcome measures for clinical trials of chronic pain treatment efficacy and effectiveness by considering the Initiative on Methods, Measurement and Pain Assessment in Clinical Trials (IMMPACT) recommendations when studying patients with chronic pain (pain, physical functioning, emotional functioning, participant ratings of improvement and satisfaction with treatment, symptoms and adverse effects and participant disposition) [120,121].

The presence of TMD can negatively influence patients’ QoL, and TMD severity determines the degree of QoL impairment [122]. A 36-item short-form (SF-36) questionnaire and oral health impact profile (OHIP-14) are considered the most popular in these studies [123]. Regrettably, only four out of 44 studies [79,89,97,98] assessed QoL, of which two of them utilised OHIP-14 [79,98], whereas the other two studies employed the anxiety assessment tool “beck anxiety inventory” (BAI) [89] and EuroQol-5D [97].

None of the included studies in this review considered utilising immunological and quantitative synovial fluid analysis, beta-glucuronidase, IgA or IgG, which demonstrates elevated levels of inflammatory mediators in diseased joints compared with asymptomatic non-diseased joints [124,125]. 

Comprehensive instruments of outcome measure assessment are vital to augment the potential of optimal clinical outcomes. Hence, this review’s authors categorised the variables’ evaluation tool instruments into quantitative and qualitative, summarised in Table S2. Additionally, the IMMPACT II tool was used to develop consensus reviews and recommendations for improving the design, execution and interpretation of clinical trials of treatments for pain [126]. This can provide insight for investigators to employ in future studies.

### 4.3. Role of Tissue Optical Properties in Determining the Therapeutic Dosimetry

Light transportation in the tissues is a complex process, owing to multiple scattering and absorption, as well to the optically anisotropic biological tissue [127]. This largely limits the ability to focus a beam into deeper tissue, yet sometimes this scattering is desired [128]. In therapeutic applications, absorption is the primary mechanism to generate physical effects, and light scattering arises from the presence of heterogeneities within a bulk medium [127]. The scattering signals can be utilised to determine optimal light dosimetry [128]. The principal parameters that affect scattering are as follows: wavelength of red to near infrared (NIR) in the range 650 to 1200 nm, relative refractive index and particle radius [129,130]. An in vivo study by Alvarenga et al. (2018) utilised semiconductor 660 nm laser irradiation trans-gingivally, which resulted in an attenuation of light intensity of 50% at 5 mm in depth [131]. Hence, a great consideration of light attenuation is transmucosal, whilst setting optimal parameters is required. In this context, all the included studies in the present review failed to consider the optical properties of the affected trigger point areas with different structural organisation, when PBM irradiation was applied with an EO or cervical (transcutaneous) or IO (transmucosal) approach or any combination. This would ultimately have a great impact on the optimal irradiation doses and a significant effect on the clinical outcome optimisation. Surprisingly, the results are positive in favour of PBMT. 

In lieu of energy loss as described, we can anticipate that the results of the included studies were either overinterpreted or underrepresented. In this context, in order to achieve standardisation and reproducibility of PBM parameters, an increase in the surface applied dose of around 10 times for sub-surface targets is recommended [132]. It is projected that at a depth of 1 cm, there will remain around 5–10% of the surface NIR photons arriving at the target tissue at that level [133,134]. 

### 4.4. Evaluation of EO (Transcutaneous) and IO (Transmucosal) PBM Therapy Approaches 

EO applications of PBM have some clinical advantages regarding convenience of use due to intraoral discomfort and treatment of a large surface area. However, the dose delivery of EO approaches as well as the treatment dosimetry to the oral tissues remain challenging and require further investigation. PBMT increases the lymphatic flow, which reduces oedema and decreases prostaglandin E2 and cyclooxygenase-2 levels [25]. Under the surface of the skin at 1 cm depth, the intensity of a laser is reduced to 10% of its value. Therefore, a laser with a power density of 100 mW/cm^2^ at the skin surface would be 10 mW/cm^2^ at 1 cm below, and at 2 cm below would be 1 mW/cm^2^ [135]. This would provide an insight in formulating the therapeutic power output and effective fluence, taking into consideration the utilised light source.

### 4.5. Evaluation of Reported Laser Treatment Parameters 

Some of the key factors determining the light depth of penetration are as follows: target optical properties (consistency, structure, thickness, skin colour, absorption/scatter coefficient), light source wavelength, shape of laser beam, duration of irradiation exposure and tissue pressure. Table 2, reported by Jenkins et al. (2011), highlights the essential and desirable laser treatments that should be reported to standardise the clinical examination of TMD patients, improve methodology reproducibility among clinicians and facilitate the comparison of results among researchers [136]. 

A further impact of the acceptance of reproducibility is the incomplete reporting of parameters in the literature. Bearing this is in mind, the authors of this review aimed to provide clinical PBM protocols guidance based on evidence derived from the literature and evidence-based and expert opinions, and is intended only for further research standpoints. We synthesised an eclectic assortment of experimental laser parameter protocols from 11 studies of this review, which utilised power meters to measure the therapeutic power output [61,65,67,70,73,75,77,83,88,89,95] as outlined below. 

#### 4.5.1. Utilisation of a Single Wavelength in Test Group

Out of the 11 studies, eight [61,65,70,73,77,83,88,89] utilised a single wavelength of red (632.8 nm), IR (808, 810 and 830 nm) or NIR (904, 910 nm) in various emission modes (CW, pulsed and super-pulsed) in the test groups, as outlined below.

Farare et al. (2008) [61] and Marini et al. (2010) [70] utilised 904 nm in CW and 910 nm in super-pulsed emission mode, respectively. Farare et al. (2008) utilised the following protocol: 904 nm, 15 mW, 7 J/cm^2^/point, 0.38 W/cm^2^/point, 16 s/point, beam area-0.039 cm^2^, in contact, twice a week for four weeks. The subjects’ symptoms in this study were associated with TMJ, temporalis and masseter muscles, as well as neck regions associated with mandibular dysfunction (restriction of MM); however, of the five TP, four were mapped in the shape of “a cross” in the pre-auricular region and the other one in the external auditory meatus, bilaterally. The authors only addressed the TMJ region but failed to address the TP of the masticatory region (IO and EO), as well the cervical muscles, knowing the patients experienced restriction in MM. Moreover, the beam area of 0.039 cm^2^ is a very small spot size to utilise for a large area to irradiate. Nevertheless, a significant reduction (*p* < 0.05) in pain level in PBM group was observed, compared to the placebo.

It is noteworthy that placebo/sham PBM, as a comparable arm in TMD study design, is essential to validate the optimal outcome [137]. 

A study by Marini et al. (2010) [70] utilised the following protocol: 910 nm, super-pulsed, 400 mW, range of 1 to 50 KHz (three steps on TMJ region: 1.2 KHz for 10 min, 1.2 KHz for 5 min and 3.16 KHz for 5 min) and pulse width of <200 ns, five days per week for two weeks (10 sessions). There were many missing data, such as dose, spot size and irradiance. Moreover, there were bilateral PBM applications on the TMJ region without specifying the number and distribution of the TP. The PBM group’s findings showed a statistically significant improvement in pain intensity on VAS and functional movements (MMO), compared to drug (ibuprofen) and sham LLLT groups at all timepoints and at the one-month follow-up. Despite this, the study showed potential in their findings but the missing data compromised the potential of its reproducibility. Only one of the included studies (power meter usage was not specified) conducted by Lassemi et al. (2008) [63], utilising 980 nm, 80 Hz and 2 J/point, reported pain reduction and jaw clicking, which were maintained up to one year, but regrettably, essential parameters such as power output, dose/point, spot size, pulse width and irradiance were unreported. 

Interestingly, a study by Emshoff et al. (2008) [65] utilised the following laser parameters: 632.8 nm, CW, 30 mW, contact, 1.5 J/cm^2^/point and 120 sec, two to three times a week for eight weeks, and their findings showed no difference between LLLT and placebo at eight weeks in reducing pain, for which LLLT was no better than the placebo at reducing pain. These findings were supported by Venacio et al. (2005) [57]. Nevertheless, taking into account the tissue optical properties, and the 632.8 nm shallow penetration depth in tissue, not surprisingly, insignificant pain reduction in the long term was noted. It is noteworthy that the following data were unreported in Emshoff et al. (2008): energy, beam area, spot size, irradiance, tissue-spot distance and neither the number of TP nor the allocations specified. Hence, it is difficult to utilise this protocol for future studies.

A study by De Carli et al. (2012) [73] used combined therapy of PBM and piroxicam and both therapies alone. The PBMT protocol was as follows: 808 nm, 2.8 J/point (total 56 J), 100 mW/point, 0.028 cm^2^, 100 J/cm^2^/point and 28 sec, twice a week (four sessions), with 30 days follow-up. Bilateral EO PBM applications of 10 TP/side were as follows: TMJ and muscle points on each side; joint capsule (lateral, posterior, superior, anterior, inferior), masseter (origin, insertion) and temporal (anterior, middle, posterior). The findings revealed a significant reduction in pain on VAS (*p* < 0.05) but combined therapies were not more effective than single therapy in TMD. This study was at a low risk of bias. Interestingly, Brochado et al. (2018) [89] utilised 808 nm as well comparing combined PBMT and manual therapy (MT) to a single therapy, with the following laser protocol: CW, 4, 48 or 576 J/point, 100 mW, contact, 0.03 cm^2^, 13.3 J/cm^2^ (total: 133 J/cm^2^), 3.33 W/cm^2^, 40 s/point, 12 TP/side and bilateral application employed (five in the TMJ region (superior, anterior, lateral, posterior and postero-inferior to the condyle) and seven for masticatory muscles (temporalis (anterior, middle and posterior), masseter (upper, middle and lower portion) and insertion of the medial pterygoid), three times a week, for four weeks (12 sessions) with various timepoints of treatment assessment (7th, 14th, 21st and 28th day) and follow-up (two and three months post-treatment). The study’s findings showed a reduction in pain in all treatment groups compared to baseline, but this was maintained only in the PBMT and PBMT and MT groups over the follow-up period. Depression and anxiety were reduced in the both the PBMT alone and PBMT and MT groups. Nevertheless, the latter did not show a superior effect, compared to PBMT alone. This study was at a low risk of bias.

Studies by Costa et al. (2017) [83] and Borges et al. (2018) [88] utilised 830 nm laser-PBM with the following protocols: 100 mW, 0.028 cm^2^, 2.8 J/point (total 14 J), five masticatory TP (EO) per side, 100 J/cm^2^, 28 s/point and single application; and 30 mW, CW, contact, 0.11600 cm^2^, four points per side (bilaterally eight points), G1: 1–8 J/cm^2^, 7.68 J and 32 s/point, G2: 57.6 J, 60 J/cm^2^ and 240 s/point and G3: 101.12 J, 105 J/cm^2^ and 420 s/point, respectively. Interestingly, in both studies there were no follow-up timepoints after the completed treatment. They only assessed the variable during the treatment duration, which was one day [83] and three weeks [88]. The findings, on the other hand, reported no statistical difference between PBM and the placebo groups in terms of passive or active mouth opening (*p* ≥ 0.05), pain improvement over the masseter muscle and total pain [83], whereas a study by Borges et al. (2018) [88] reported pain reduction in all the groups; however, a statistically significant increase in MOM and an improvement in mandibular protrusion was only observed with fluence values of 1–8 J/cm^2^. The latter study is of significance, as it tested three PBM doses to identify the optimal results’ values. This study’s drawback was a lack of follow-up timepoints to determine the long-term effects of PBMT. In the context of PBM dose, a study by da Silva et al. (2012) [74] (power meter usage was not specified) tested two fluence values (780 nm, 70 mW, CW, contact, beam diameter—5 mm, GI: 52.5 J/cm^2^, 30 s, GII: 105 J/cm^2^, 60 s, twice a week for five weeks) to identify optimal clinical outcome compared to the placebo. The findings show a statistically significant reduction in pain on VAS and an increase in the mandibular range of movements, favouring a high fluence of 105 J/cm^2^; the drawback of this study was a lack of follow-up after the completed treatment.

#### 4.5.2. Utilisation Two Laser Wavelengths in Test Group

One study out of 11 conducted by Shirani et al. (2009) [67] used two combined laser wavelengths; 660 and 890 nm, in CW and pulsed mode, respectively, in the test group, compared to the placebo, based on the following laser protocols: spot size 0.6 cm^2^, 1 cm^2^ irradiated area, extraoral masseter muscle and intraoral MPM and LPM TP (number and distribution of TP unspecified), in contact, mild tissue pressure applied, twice a week for three weeks (six sessions in total), 660 nm (CW, 6–10 J/point, 17.3 mW, six mins exposure time) and 890 nm (pulsed, 1500 Hz, 9.8 W, 1 J/cm^2^, 10 min exposure time). In this study, the recruited subjects were only females with a mean age of 23.8 years old. Positive results were reported in using combined GaAs and InGaAlP lasers, usually applied for deep-lying disorders and superficial disorders, respectively, for pain reduction, compared to placebo. This was supported by a study conducted by Wang et al. (2011) [138] applying GaAlAs at 650 and 830 nm, implying that the combination of two laser wavelengths may be beneficial to patients with TMD.

Based on the above notes, red wavelengths are more easily absorbed by tissue surface components than infrared wavelengths. The absorbed energy can be dissipated in the form of heat around the skin surface. Conversely, the infrared wavelengths can penetrate deeply into the body tissues, which have lower scattering and absorption properties [139]. In this context, different wavelength utilisation, red and infrared radiation, can act on different sites of the tissue. Red light acts on the mitochondria, whereas IR acts on both mitochondrial and cellular membranes [140]; the combined effects of both wavelengths can be advantageous for tissue biomodulation. Future studies should be performed combining red and infrared radiation for TMD treatment. 

#### 4.5.3. Mixed light Sources in the Test Groups

Out of the 11, two studies [75,95] utilised the following various light sources in the test groups. A study by Panhoca et al. (2013) [75] employed the following protocols for three recruited groups: red (630 ± 10 nm) (9 J/point,18 J/cm^2^,150 mW, 300 mW/cm^2^/point, 0.5 cm^2^), IR (850 ± 10 nm) LEDs, (9 J/point, 18 J/cm^2,^ 150 mW, 300 mW/cm^2^/point, 0.5 cm^2^) and IR laser (780 nm) positive control (4.2 J/point, 70 mW, 0.04 cm^2^, 1.7 W, 7 cm^2^/point, 105 J/cm^2^), 60 s/point exposure time, five EO TP and contact, twice a week for eight weeks (total of eight sessions). A significant reduction in pain and an increase in MOM for all groups (*p* < 0.05) was reported. There was no significant difference in the pain scores and maximum oral aperture between groups at baseline or any periods after treatment (*p* ≥ 0.05). All the fluences of 18 and 105 J/cm^2^ were shown to be effective. Interestingly, Panchoca et al. (2015) [75] chose the applied dose based on typical doses used in regular practice of around 100 J/cm^2^, which was shown to increase the muscle activity when irradiated with LED PBM therapy compared with the placebo. In this context, a study by Pöntinen et al. (2000) showed that a fluence of 4 J/cm^2^ at skin level has maintained irradiance at depths in the range of 0.5 to 2.5 cm [141]. Following irradiation of joints or muscles, a fluence of 100–300 J/cm^2^ was attenuated to 2 J/cm^2^, and irradiance can be maintained at certain depths [141,142].

A recent study by Herpich et al. (2020) [95] utilised a nine-clusters head of the following various light sources: one super-pulsed diode laser (905 nm), four red LEDs (640 nm) in pulsed mode and four IR LEDs (875 nm) in pulsed mode (Table S1). This study aimed to assess the influence of an intraoral approach of PBMT in a female cohort (30 in total with mean age of 31.7 ± 5.2 years old). Significant differences between groups were found regarding pain (*p* ≤ 0.01) and functioning (*p* ≤ 0.04), compared to sham. However, no statistically significant difference was found regarding range of mandibular motion (*p* > 0.05). This implies the advantages of utilising combined laser and LED PBM of red and IR, with different emission modes of super-pulsed and pulsed, in patients with TMD. Important to note is that the aperture size measured 4 cm^2^, but an adapter with an aperture of 0.394 cm^2^ was used for IO application.

As all the included studies did not specify the beam profile, we assume it is Gaussian, which is of a very small spot size, and the distribution of the energy is uneven at the peripheral area, receiving <50% of that at the centre. To overcome this challenge, PBM handpieces were developed to optically correct the beam profile to a flat-top shape with colimitation distribution, an evenly uniform delivered energy over 1 cm^2^ [132]. 

The above included studies [61,65,67,70,73,75,77,83,88,89,95] in this review made efforts in utilising the power meter to determine therapeutic power output to achieve consistent and optimal outcomes; however, they have proven elusive, due to the diversity of their findings. However, the dual wavelengths and the use of laser and LED light sources have provided a perspective for suggested clinical guidance for future extensive studies, taking into consideration the necessity for the development of LED and laser prototype devices with the same geometry and parameters, in order to achieve standardisation and results validation.

Based on the above-gathered notes, the authors have answered the focused review questions and have proposed suggested recommendations for clinical PBMT protocols for future extensive TMD RCT studies, which are grounded in the current available evidence-based clinical practice and experts in the field (Table 3). Additionally, a summary of the influencing factors that are important for TMD study design and recommendations suggested for future reproducible methodology are illustrated in Table 4. The latter would ultimately assist in paving the roadmap for scientific consensus in TMD management. 

#### 4.5.4. Evaluation of the Trigger and Palpable Points and Applications of Irradiation

It is very clear from our results that there are inconsistencies and discrepancies in the number, allocation and distribution of TP among the majority of the included studies. Additionally, there is a lack of consideration of the cervical muscles, such as SCM, trapezius and belly of the digastric muscles noted, despite the fact there is a correlation between the level of tenderness of masticatory and cervical muscles and jaw dysfunction and neck disability [143,144]. Disability has a great impact on patients’ QoL [145]. Hence, irradiation of the cervical muscles needs to be part of the TMD PBM irradiation protocol.

Importantly to note, bilateral PBM irradiation, especially in chronic TMD pain, regardless of unilateral or bilateral TMD symptoms presentation, is crucial to optimise clinical outcomes and prevent relapse due to the compensation phenomena [146]. In our review, only 21 out of 44 eligible studies reported bilateral applications of the PBMT protocol [57,58,60,61,64,70,73,74,75,76,77,80,81,83,86,88,89,90,95,97,100], whereas two studies specified a unilateral PBMT application protocol [72,92]. The remaining 21 studies failed to specify. Hence, inconsistency was noted in the reported number of studies’ results. 

**Figure 13:**
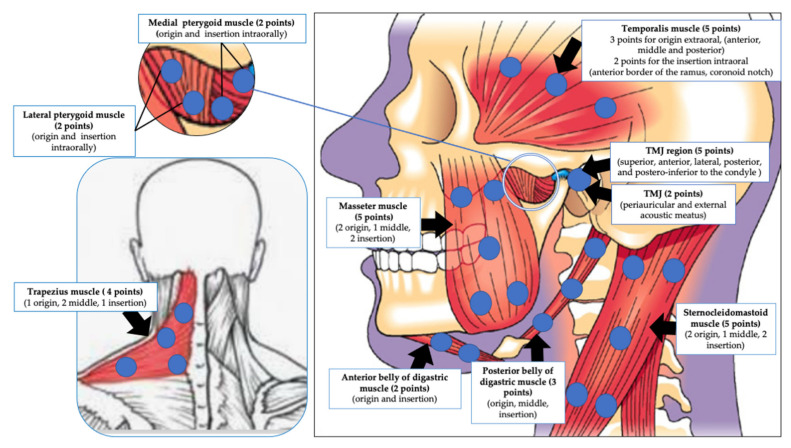
Schematic representation of the proposed suggested number and allocations of the trigger points for PBM irradiation in TMD management. They are based on evidence derived from the literature and expert opinion and are intended only to provide clinical guidance and serve as a starting point for extensive research. The blue circle represents the trigger points’ allocations and their number.

Based on the above notes, the authors of this review have extrapolated from this review’s findings the required TP and their allocations for PBM irradiation to be considered, as a guide for future extensive studies (Figure 13).

### 4.6. Role of RoB Assessment

Risk of Bias assessment revealed that 38% studies reported a low risk of bias, while 46% studies were at high risk and 16% studies had some concerns. The majority of the high risk of bias in the quality of available evidence came from a randomisation process (12 out of 44 studies) [58,60,62,76,77,78,80,84,85,86,87,98], deviations from intended interventions (18 out of 44 studies) [58,59,62,63,76,77,78,80,81,84,85,86,87,88,91,95,96,99] and measurement of the outcome (12 out of 44 studies) [59,63,75,76,78,80,84,86,87,96,97,99]. 

The results of this meta-analysis show that in terms of reducing pain intensity and improvement in mouth opening, PBMT shows statistically significant superior results in comparison to control intervention groups. Presence of a potential conflict of interest was observed in two out of 44 studies [59,95]. 

### 4.7. Role of Meta-Analysis Outcome

The present systematic review and meta-analysis was based on the hypothesis that PBMT with LEDs or lasers is as beneficial as or superior to placebo (sham PBM), the pharmacological approach, the cognitive approach, physiotherapy, conservative treatment modalities (occlusal splint), ultrasound, TENS, alpha-lipoic acid or needle therapy, in terms of pain intensity reduction (chronic pain), functional improvement (masticatory malfunction) or anxiety/depression improvement, or QoL in patients with TMD. Consequently, a critical appraisal of the available scientific evidence was conducted. Meticulous scrutinisation of the available scientific literature resulted in the inclusion of 44 studies in this systematic review and meta-analysis [57,58,59,60,61,62,63,64,65,66,67,68,69,70,71,72,73,74,75,76,77,78,79,80,81,82,83,84,85,86,87,88,89,90,91,92,93,94,95,96,97,98,99,100]. 

Owing to insufficient numerical data and methodological discrepancies, only 32 out 44 studies qualified for a meta-analysis [57,58,60,61,62,63,64,65,66,67,68,69,70,71,72,73,74,77,78,81,83,84,85,86,88,89,91,95,96,97,99,100]. Consequently, a meta-analysis of 30 out of 32 studies was performed on pain reduction assessment by qualitative measurement with VAS, which showed a statistically significant improvement in the PBMT group, as compared to the control group with high heterogeneity [57,58,60,61,62,63,64,65,66,67,68,69,70,71,73,74,78,81,83,84,85,86,88,89,91,95,96,97,99,100]. A subgroup analysis on the role of length of follow-up evaluation in pain reduction assessment with VAS revealed that the length of follow-up evaluation was not a contributing factor towards the highly heterogenous results obtained in the abovementioned meta-analysis. Hence, a sensitivity analysis after elimination of 16 high-risk outlier studies identified by RoB assessment and a visual examination of the Forest plot summary [58,62,63,65,67,71,78,81,84,85,86,88,91,96,97,99] was performed, which then revealed results favouring the PBMT group, with low heterogeneity in the presented data. 

Additionally, a meta-analysis on four out of 32 studies of assessment of PPT levels [69,71,72,81] and 11 out of 32 studies of MMO assessment [58,70,73,74,77,83,84,85,95,96,100] was performed, which showed statistically significant improvement in the PBMT group, compared to the control group with low and no signs of heterogeneity, respectively. No significant risk of publication bias for pain reduction assessment by VAS, PPT and functionality improvement assessment by MMO was noted in this meta-analysis. 

QoL is an important tool for assessment of how an individual’s emotional, social and physical well-being are affected by disease/disorder/disability in their daily life [147]. However, only four out of 44 studies [79,89,97,98] assessed this parameter with heterogeneity in the presented data, thereby resulting in a lack of a possible meta-analysis to assess QoL.

Petrucci et al. (2011) conducted a systematic review and meta-analysis on six RCTs to assess the efficacy of LLLT in the treatment of TMD [31]. The meta-analysis findings revealed no statistically significant difference in pain reduction assessment by VAS for the LLLT group as compared to the placebo [147]. A systematic review and meta-analysis by Chang et al. (2014) assessed the use of LLLT on the masticatory muscle or joint capsule for TMJ pain in seven randomised, single- or double-blind studies and showed that the former had a moderate analgesic effect in comparison to placebo/sham LLLT [148]. However, the authors were unable to deduce an optimal LLLT protocol to treat TMJ pain [148]. Chen et al. (2015) conducted a meta-analysis of 14 randomised controlled trials and evaluated the effectiveness of LLLT for patients suffering from TMDs, and concluded that in comparison to the placebo, LLLT was unable to reduce chronic TMD pain, but provided a significantly better functional improvement in comparison to the placebo [118]. Xu et al. (2018) conducted a systematic review and meta-analysis to evaluate the effect of LLLT vs. placebo in 31 RCTs performed on patients with TMD [149]. 

In the present systematic review and meta-analysis is an up-to-date appraisal of the available scientific literature with a robust search strategy ranging from 2005 to 2021, resulting in the inclusion of a large cohort of studies. The results of this review show that PBMT effectively relieves pain assessment by VAS and improves the functional outcomes in patients with TMD at the short-term follow-up [118]. The authors also performed dosage analysis, which showed inconsistent results regarding the effects of high or low doses for patients with TMD [118]. The results of our systematic review and meta-analysis contradict the findings of Petrucci et al. (2011) [31] and Chen et al. (2015) [118] (pain reduction assessment by VAS); however, they are in accordance with Chang et al. (2014) [148], Chen et al. (2015) [118] (functional improvement) and Xu et al. (2018) [149].

## 5. Conclusions and Future Direction

To date, this is the first extensive systematic review and meta-analysis (44 eligible RCTs, of which 32 studies were eligible for meta-analysis) synthesising an eclectic assortment of experimental protocols. The majority of the available evidence suggests that PBM of laser or LEDs, or combined treatment modalities, have fundamental and substantial effects on improving TMD chronic pain, functionality and QoL. Notably, both red light and NIR, as well red/NIR combination light sources, were utilised to target superficial and deep target tissues, assisting to improve pain and functionality. Despite the heterogeneity of the eligible studies’ clinical outcomes, the majority of them were of low-risk bias. Hence, researchers must hone in on specific clinical PBM protocols, therapeutic outcome measures, underlying mechanisms and replicability. Hence, for the first time, suggested recommendations for clinical PBMT protocols for future extensive TMD RCT studies were proposed by this review’s authors, grounded in the current available evidence-based clinical practice and experts in the field, as well as a summary of TMD guidance study design for future reproducible methodology, which is ultimately the first steppingstone for evidence-based consensus. Additionally, there is still a considerable gap between the potential findings of PBMT in TMD management and understanding their underlying biological and molecular mechanisms, which require the scientific community’s attention.

## Figures and Tables

**Table 1:** The qualitative and quantitative measurements for primary and secondary outcomes utilised in the selected studies of this review, with consideration for TMJ synovial fluid analysis to evaluate the levels of oxidative stress, IL-1,6,8, TNF-α and β, MM-1,2,9, VEGF, TGF-β1 and IGF-I, for future extensive research. All the abbreviations in this table are listed in Supplementary File S2.

Assessment of Outcome Measures	Primary Outcomes	Secondary Outcomes	
	Pain Reduction	Functional Improvement	Anxiety/Depression and QoL
**Qualitative** **(patient-reported ** **outcomes; subjective)**	Visual analogue scale (VAS) Numerical scale of pain OHIP/TMD questionnaire McGill pain questionnaire Symptom severity index Orofacial myofunctional evaluation protocol with scores (OMES)	Patient-specific functional scale	Euro Qol-5D 5L Beck anxiety inventory (BAI) Pain distress scale
**Quantitative** **(objective)**	Kaplan–Meier method Pressure pain threshold (PPT) (dial algometer) Power algometer	Kaplan–Meier method Jaw kinesiology Craniomandibular index (CMI) Colorimetric capsules Electromyography (EMG) Digital pachymeter (Digimess) to measure the vertical and horizontal movements Stethoscope (crepitation) Helkimo index Anamnestic questionnaire Computerized photogrammetry Masticatory test Active range of motion (AROM) index Digital calliper Flexible millimetre ruler Millimetre ruler RDC/TDM	
**TMJ Synovial fluid analysis** **(immunological** **profile)**	To evaluate the levels of the following data: oxidative stress, IL-1,6,8 (Interleukin-1,6,8), TNF-α (Tumor necrosis factor- alpha) and β, MM-1,2,9 (Mandibular movement-1,2,9), VEGF (Vascular endothelial growth factor), TGF-β1 (Transforming growth factor-β1), IGF-I (Insulin-like growth factor-I)		

**Table 2:** The essential and desirable PBM parameters that should be reported to standardise the laser protocol for TMD patients, improve methodology reproducibility among clinicians and facilitate the comparison of results among researchers [136]. All the abbreviations in this table are listed in Supplementary File S2.

Essential Reported Parameters			Desirable Reported Parameters
Device Information	Irradiation Parameters	Treatment Parameters	Energy per Pulse (J)
Manufacturer	Wavelength (nm)	Beam spot size at target (cm^2^)	Polarisation
Model identifier	Spectral bandwidth (nm)	Irradiance at target (mW/cm^2^)	Aperture diameter (cm)
Emitters type (e.g., nGaAlP LED, GaAlAs LASER, KTP LASER)	Operating mode (CW, pulsed, super pulsed)	Exposure duration (sec)	Irradiance at aperture (mW/cm^2^)
Number of emitters	Frequency (Hz)	Radiant exposure (J/cm^2^)	Beam divergence (°)
Spatial distribution of emitters. (e.g., 4 emitters spaced 2 cm apart in a square pattern).	Pulse width (second)	Radiant energy (J)	Beam shape
Beam delivery system (e.g., fibreoptic, free air/scanned, hand-held probe).	Duty cycle (%)	Number of points irradiated	Scanning technique
	Beam profile	Area irradiated (cm^2^)	Speed of movement
		Application technique	
		Number and frequency of treatment sessions	
		Total radiant energy (J)	

**Table 3:** Illustrates PBMT protocols for chronic TMD symptoms of deep-seated tissue injurie derived from the literature and evidence-based clinical practice (studies utilised power meter) and expert opinion. These protocols are intended only to provide clinical guidance and to serve as a starting point for extensive research. ** All the abbreviations in this table are listed in Supplementary File S2.

Delivery Route of PBM Irradiation **	Affected Regions **	Treatment Area and No. of TP (Optimal Target Tissue), Depending on Palpable Areas, Including Cervical Muscles ** (Figure 13)	PBM Device Characteristics, Application and Treatment Protocol **	Frequency and Treatment Duration Protocol **
**Extraoral (EO)**	TMJ	External acoustic meatus: 1 Periauricular: 1	**Laser λ: 790–830 nm** [65,73] Therapeutic power output (at the target): 100–500 mW; emission mode: CW; irradiated area: 0.5–1 cm^2^ Contact on skin surface Exposure time: 30–60 s A firm pressure on skin surface applied to increase light penetration depth **Laser and LEDs**: [75] Red (630 ± 10 nm) (9 J/point, 18 J/cm^2^, 150 mW, 300 mW/cm^2^/point, 0.5 cm^2^), IR (850 ± 10 nm) LEDs, (9 J/point, 18 J/cm^2^, 150 mW, 300 mW/cm^2^/point, 0.5 cm^2^) IR laser (780 nm); positive control (4.2 J/point, 70 mW, 0.04 cm^2^, 1.7 W/cm^2^/point, 105 J/cm^2^), Fluence: 18 and 105 J/cm^2^	2–3 times a week At least 4 consecutive weeks (total of 8–10 sessions), depending on status of presented symptoms (acute or chronic)
	TMJ-associated regions	Superior, anterior, lateral, posterior, postero-inferior to the condyle: 5		
	Masticatory muscles	Temporalis: 3 Masseter muscle: 5 LPM: 2, MPM: 2		
	Cervical muscles	Trapezius: 4 Sternocleidomastoid muscle: 5 Anterior belly of digastric muscle: 2 Posterior belly of digastric muscle: 3		
**Intraoral (IO)**	Masticatory muscles	Superficial head of the MPM: 1 LPM: 1 Insertion of temporalis muscle: 2	**Laser (905 nm) and LEDs (4 clusters of 640 nm (Red) + 4 clusters of 875 nm (IR))** [95] 905 nm diode laser: 0.9 mW (MOPO), 1000 Hz, 0.4 cm^2^, 300 s, super-pulsed 640 nm LED: 15 mW (MOPO), 2 Hz, 0.9 cm^2^, 300 s, pulsed 875 nm LED: 17.5 mW (MOPO), 16 Hz, 0.9 cm^2^, 300 s, pulsed Adapter with aperture of 0.394 cm^2^ Dose: 99.67 J/cm^2^/point	
**Combined IO and EO**	Superficial head of the MPM: 1 (IO) LPM:1 (IO) Insertion of temporalis muscle: 2 (IO) Masseter muscle: 5 (EO)		**Laser: combined λ 660 + λ 890 nm** [67] 660 nm (CW, 6–10 J/point, 17.3 mW, 360 s exposure time), 890 nm (pulsed, 1500 Hz, 9.8 W, 1 J/cm^2^, 600 s), mild tissue pressure applied, irradiated area 0.6 cm, dose: 6–10 J/cm^2^ (deep-seated tissue) and 1–4 J/cm^2^ (superficial-seated tissue)	

**Table 4:** Illustrates summary of the key factors that are important in TMD study design and suggested recommendations for reproducible methodology, intending only to provide clinical guidance and serve as a starting point for extensive research. ** All the abbreviations in this table are listed in Supplementary File S2.

Key Factors **		Suggested Recommendation **	Description and Citation of Scientifc Evidence ** (Manuscript)
**Population characteristics**	Age range	*Paediatric cohort:* <18 years old *Adult cohort:* 18–40 years old 41–60 years old >60 years old	Refer to Section 4.1
	Gender	Either female or only male category for each study. Mixed-gender subjects, depending on aims and objectives of the study	
	Racial background	It is noteworthy that skin colour plays an important role in the scattering and absorption of the photonic energy. It can have a great impact on PBM dosimetry	
	Optical properties of the target tissue	Identify the consistency, structure, thickness, skin colour and absorption/scattering coefficient of the target tissue	Refer to Section 4.3
	Sample size	Even distribution of the sample size to the intervention and placebo groups Same gender cohort in each study Same racial background cohort in each study and avoid mixed racial background in each study Sample size should be at least 25 in each group	Refer to Section 4.1 and Section 4.2.1
**Randomisation and blinding processes**		Two independent blind investigators to assess the variables at all timepoints (double-blind) and record the data. Robust randomised process. Parallel arm study design.	Refer to Section 4.2.1
**Comparable arms of the study**		Placebo/sham PBM, as a comparable arm in TMD study design, is essential to validate the optimal outcome. It assists in providing standarised and reproducible data	[137]
**Presented symptoms**	**Pain**: TMJ: pain, arthralgia TMJ-associated areas EO or IO masticatory muscles (or combination) Cervical muscles A combination of any of the above areas, depending on the presented symptoms **Functional disability:** Limited mouth movements, highlighting the degree of severity. Difficulty in chewing TMJ clicking/or crepitation Jaw protrusion or deviation, during mouth opening or closing Cervical muscles stiffness **Anxiety/depression**	- Diagnosis of the symptoms, whether they are acute or chronic, as they have a great impact on the laser treatment protocol - Thorough clinical examination to identify the palpable TP (EO and IO), along with the unpalpable TP, which can contribute to TMD symptoms such as tenderness in the cervical muscles - Identifying functional disabilities and their contributions to TMD symptoms - Pre-treatment measurement of mouth opening by 2 independent investigators, utilising 1 or 2 of the assessment tools reported in Table S2 - Assessment of a patient’s anxiety/depression is crucial for all TMD patients, as a routine assessment measure	Refer to Section 4.2.1 and Table S2
**Diagnostic criteria**		Combining 2 tools: RDC/TMD and diagnostic manual muscle testing (MMT) for cervical muscles assessment	Refer to Section 4.2.2
**Standardised laser protocol**		Based on gathered evidence-based practice and science, 11 out of 44 studies utilised power meter and placebo/sham PBM; recommendations of PBMT protocols suggested for further research	Refer to Table 4 and Section 4.5
**Light device standardisation**		Standardised prototype development for each involved light source in the study	
**Route of delivery of PBM irradiation**		Identifying the consistency, structure, thickness, skin colour and absorption/scattering coefficient of the target tissues (optical properties) of each of the following PBM delivery rout(s), prior to setting up the PBM parameter protocols: Transmucosal approach (IO) Transcutaneous approach (EO) Combination of the above approaches	Refer to Section 4.4 and Table 4
**Unilateral/bilateral PBM irradiation**		Regardless of unilateral or bilateral TMD symptoms, PBM irradiation needs to be applied bilaterally, due to the concept of compensation effects	[147]
**Trigger** **Points (TP)**	Affected sites Number of the TP/site	All the TP need to be addressed as follows: EO and IO muscles’ contribution to TMD, including the masticatory muscles, as well as the cervical (palpable or unpalpable). The number of the TP depends on the origin and insertion of each muscle, its location and volume	Refer to Section 4.5.4 and Figure 13
**The investigated variables and follow-up timepoints**		- Pain - Functional problems - Anxiety/depression - QoL Address these variables at T0 (pre-treatment), mid-treatment and end-treatment, then 1, 3, 6, 12 and 18 months post treatment	Refer to Section 4
**Outcome measures**		- Qualitative or quantitative or combination assessment tools (Table S2) - Including the synovial fluid assessment is vital, which has not been employed in all the eligible studies of the present review. Therefore, immunological and quantitative synovial fluid analysis, beta-glucuronidase, IgA and IgG demonstrate elevated levels of inflammatory mediators in diseased joints compared with asymptomatic non-diseased joints [124,125] - IMMPACT recommendations [126] - Utilise QoL index	Refer to Table S2 and these citations: [124,125,126]
**Reported data**		Documentation of essential and desirable PBM parameters is pivotal for reproducibility and standardisation	Refer to Table 2 [135]

## Supplementary Materials

The following are available online at https://www.mdpi.com/article/10.3390/antiox10071028/s1, Table S1: Tabular description of all the selected eligible in vivo RCTs human studies of TMD, in terms of demography, study design, symptoms, affected areas, functional problems, intervention groups, methods of assessment, evaluation period and outcomes. All the abbreviations in this table are listed in Supplementary File S2; Table S2: Tabular representation of lasers/LEDs parameters utilised in the chosen eligible in vivo RCTs human studies related to temporomandibular dysfunction syndrome (TMD). All the abbreviations in this table are listed in Supplementary File S2; Table S3: Tabular description of all the selected eligible in vivo RCTs human studies of TMD, in terms of level of significance in subjective and objective assessments of pain, functionality improvement and anxiety reduction/QoL improvement. The abbreviations are in this table are listed in Supplementary File S2; Supplementary File S1: PRISMA checklist; Supplementary File S2: List of abbreviations.
