# Supplementary material for: Role of Photobiomodulation Therapy in Modulating Oxidative Stress in Temporomandibular Disorders. A Systematic Review and Meta-Analysis of Human Randomised Controlled Trials
Source: Antioxidants (Basel). 2021 Jun 25;10(7):1028. doi: 10.3390/antiox10071028 (PMC8300797; doi:10.3390/antiox10071028)
Supplement: Supplementary file 1 [file antioxidants-10-01028-s001.zip › supplementary-2021.7.1/Supplementary File S2.pdf]

Supplementary File S2: List of abbreviations (In alphabetical order)

Sr. no.	Abbreviation	Full form
1.	AROM	Active range of motion reference
2.	ATP	Adenosine triphosphate
3.	BD	Twice a day
4.	CCO	Cytochrome c oxidase
5.	CG	Control group
6.	COX-2	Cyclooxygenase- 2
7.	CCRCT	Cochrane Central Register of Controlled Trials
8.	CCM	colorimetric capsule method
9.	CBP	computerised biophotogrammety
10.	CI	Confidence interval
11.	cm	Centimetre
12.	cm^2	Square centimetre
13.	CW	Continuous emission mode
14.	DA	Digital algometer
15.	DF	Degrees of freedom
16.	↓	Decrease
17.	et al	And others
18.	EO	Extraoral
19.	EMG	Electromyogrpahy
20.	EuroQol-5D	European Quality of life-5 dimentions
21.	F.n.	Fusobacterium nucleatum
22.	GaAlAs	Gallium-aluminium-arsenide laser
23.	GaAs	Gallium-arsenide laser
24.	Ga-Ar	Gallium argo
25.	GMD	Geometric mean diameter
26.	GI	Group I
27.	GII	Group II
28.	GIII	Group III
29.	GIV	Group IV
30.	GV	Group V
31.	G1	Group 1
32.	G2	Group 2
33.	G A	Group A
34.	G B	Group B
35.	G 1	Group 1
36.	G 2	Group 2
37.	HeNe	Helium-neon laser
38.	HA	Hyaluronic acid
39.	hr	Hour
40.	↑	Increase
41.	IL	Interleukin
42.	IL-1β	Interleukin-1 beta
43.	IL-1,6,8	Interleukin-1,6,8
44.	IF-γ	Interferon- gamma

45.	IF	Impact Factor
46.	IgA	Immunoglobulin A
47.	IGF-I	Insulin-like growth factor-I
48.	IgG	Immunoglobulin G
49.	IO	Intraoral
50.	IR	Infrared
51.	Inf.	Inferior
52.	InGaAlP	Indium-gallium- aluminum-phosphide laser
53.	IMMPACT II	Initiative on Methods, Measurement, and Pain Assessment in Clinical Trials II
54.	I^2	Statistical test for percentage variation due to heterogeneity
55.	J	Joule
56.	J/cm^2	Joules per square centimeter
57.	KMM	Kaplan-Meier method
58.	LPM	Lateral pterygoid muscle
59.	LG	Laser group
60.	LEDs	Light emitted diodes
61.	LLLT	Low level laser therapy
62.	MHI	Maximum habitual intercuspation
63.	NRS	Numeric Rating Scale
64.	MP	Mandibular protrusion
65.	MT	Manual therapy
66.	MPM	Medial pterygoid muscle
67.	MeSH	Medical Subject Headings
68.	MVC	Maximal volunteer clenching
69.	min	Minute
70.	µm	Micrometer
71.	µsec	Microsecond
72.	Msec	Millisecond
73.	mm	Millimeter
74.	MM	Mandibular movement
75.	MOPO	Mean optic power output
76.	MO	Mouth opening
77.	MMO	Maximum mouth opening
78.	MMP-1	Matrix metalloproteinase-1
79.	MMP-2	Matrix metalloproteinase-2
80.	MMP-7	Matrix metalloproteinase-7
81.	MMP-9	Matrix metalloproteinase-9
82.	MP	Masticatory performance
83.	MRI	Magnetic resonance imaging
84.	MTP	Myofascial trigger point
85.	mW	Milliwatt
86.	M/F	Male/ Female
87.	n	Sample size
88.	N	No
89.	No.	Number
90.	nsec	Nanosecond
91.	nm	Nanometer
92.	NI	No information
93.	NA	Not applicable

94.	Nd:YAG	Neodymium-doped yttrium aluminum garnet
95.	NS	Not specified
96.	OTM	Optical test material
97.	OS	Oxidative stress
98.	OSG	Occlusal splint group
99.	PBM	Photobiomodulation
100.	PBMT	Photobiomodulation therapy
101.	PGE 2	Prostaglandin E2
102.	PG	Parallel group
103.	PI	Pain intensity
104.	Post.	Posterior
105.	PPT	Pessure pain threshold
106.	PRISMA	Preferred Reporting Items for Systematic Reviews and Meta-Analyses
107.	PROSPERO	Prospective Register Of Systematic Reviews
108.	Q	Cochran's heterogeneity statistic
109.	QoL	Quality of life
110.	ROS	Reactive oxygen species
111.	RoB 2	Revised Cochrane Risk-of-Bias tool for Randomized trials, Version 2.0
112.	RCT	Randomised clinical trial
113.	RDR	Researcher-developed rule
114.	RDC/TMD	Research Diagnostic Criteria for Temporomandibular Disorders
115.	SCM	Sternocleidomastoid muscle
116.	SD	Standard deviation
117.	SE	Standard error
118.	SMD	Standardised mean difference
119.	sec/ s	Second
120.	SS	Statistical significant
121.	Submand.	Submandibular
122.	Sup.	Superior
123.	TENS	Transcutaneous electrical nerve stimulation
124.	TGF-β1	Transforming growth factor-β1
125.	TMJ	Temporo-mandibular joint
126.	TMD	temporomandibular disorder
127.	TNF-α	Tumor necrosis factor- alpha
128.	TNF-β and α	Tumour necrotic factor beta and alpha
129.	TP	Trigger points
130.	VAS	Visual analogue scale
131.	VEGF	Vascular endothelial growth factor
132.	W	Watt
133.	W/cm^2	Watts per square centimetre
134.	WBG	Willis Bite Gauge
135.	Y	Yes
136.	%	Percentage
