# Supplementary material for: Role of Photobiomodulation Therapy in Modulating Oxidative Stress in Temporomandibular Disorders. A Systematic Review and Meta-Analysis of Human Randomised Controlled Trials
Source: Antioxidants (Basel). 2021 Jun 25;10(7):1028. doi: 10.3390/antiox10071028 (PMC8300797; doi:10.3390/antiox10071028)
Supplement: Supplementary file 1 [file antioxidants-10-01028-s001.zip › supplementary-2021.7.1/Table S1.pdf]

Table S1. Tabular description of all the selected eligible *in vivo* RCTs human studies of TMD, in terms of demography, study design, symptoms, affected areas, functional problems, intervention groups, methods of assessment, evaluation period and outcomes. All the abbreviations in this table are listed in Supplementary File S2.

Study, year, origin & citation	Journal name/IF	Cond-ition type	Presented symptoms & duration	Affected area (s)	Functionality problem	Sample size	Gender M/F	Age (yrs) (mean ± SD)	Skin colour	Intervention groups	Evaluation period	Assessed parameters	Evaluation methods	Conclusion
Venancio et al., 2005, Brazil [57]	Journal of Oral Rehabilitation IF 2021: 2.304 IF 2005: 0.904	TMD	TMJ pain & mandibular dysfunction/ capsulitis/ synovitis & disk displacement + reduction 6 months	TMJ	TMJ pain	30	GI: 2/13 GII: 3/12	GI: 34.9 (15–36) GII: 37.6 (13–63)	NI	GI: LILT GII: Sham laser (placebo).	A baseline, 15th 30th & 60th days	Pain assessment, range of MM	VAS, MM (electronic digital calliper)	SS in pain reduction on VAS (*p*=0.2060), MVO (*p*=0.3024) & PPT (*p*>0.05) in both GI&II
Çetiner et al., 2006, Turkey [58]	Photomedicine, and Laser Surgery (Now: Photobiomodula-tion, Photomedicine, and Laser Surgery) IF 2019: 1.918 IF 2006: 1.455	TMD	Myogenic TMD-associated orofacial pain, limited MM chewing difficulties, and tender points	Joint capsule (lateral post., sup.), masseter (ant., inf., deep), temporal (ant., deep, middle, origin), MPM, LPM [bilaterally]	Orofacial pain, limited MM chewing difficulties	39 G1: 24 G2:15	G1: 2/22 G2: 2/13	G1: 33.8 ±13.9 (16–62) G2: 29.6 ± 12.5 (17–50)	NI	G1: LLLT G2: Placebo	At baseline , immediately & 1 month after therapy	Pain, MMO, chewing difficulty	VAS, MMO (millimetre ruler), Lateral MM	SS reduction in PI & chewing difficulties in G1 were just after & 1- month after treatment, compared to G2 (p<0.05). No. of TP decreased significantly in G1 (*p*<0.05). No significant in the mean difference parameters after the therapy and 1 month later in G1&2 (*p*>0.05). SS in MMO between G1& G2 at all follow-up periods.
Fikackova et al., 2007, Czech Republic [59]	Photobiomodula-tion, Photomedicine, and Laser Surgery IF 2019: 1.918 IF 2007: 1.207	TMD	Myofascial pain and TMJ arthralgia	TMJ	Myofascial pain	80 GA: 33 GB: 28 GC: 19	9/71	41 (16–70)	NI	GA: 10 J/cm² GB: 15 J/cm² GC: 0.1 J/cm²	At baseline.2nd day & last (10th day) LLLT session	Pain	Pain assessment	SS in PI reduction in GA&B than GC (*p*=0.002). No significant intergroup differences & this applied to myofascial pain and/or TMJ arthralgia. SS in TMD duration in GA&GB more than GC (*p*=0.0003), no significant difference between GA &GB.
Mazzetto et al., 2007, Brazil [60]	Cranio: the Journal of Craniomandibul-ar Practice IF 2019: 1.186 IF 2007: 0.691	TMD	Capsulitis, synovitis, retrodiscitis & painful disk displacement with reduction.	TMJ	Pain	48	NI	> 18	NI	GI:LLLT GII: Placebo	Ev1: baseline Ev2: 4th LLLT application Ev3: 8th LLLT application	Pain assessment	VAS	A decrease in PI at 2nd (mean=2.93), 3rd (mean=2.236) & 4th (mean=2.583) evaluations, independent of probes & palpation, being more evident from 3rd

											Ev4: 30 days after last application			evaluation after 8th application.
Frare et al., 2008, Brazil [61]	Brazilian Journal of Physical Therapy IF 2019: 2.1 IF 2008: 0.154	TMD	Pain, joint cracking, temporal, masseter & cervical pain, tooth wear/ fracturing & joint rigidity,	TMJ, temporal, masseter muscle and neck region	Pain, joint cracking, muscular tension & joint rigidity	18 G1: 10 G2:8	0/18	18-45	NI	G1: LLLT G2: Placebo	At baseline, immediately after each session for 4 weeks.	Pain assessment	VAS	SS reduction ($p<0.05$) in PI in G1 compared to G2.
Da Cunha et al., 2008, Brazil [62]	International Dental Journal IF 2019: 2.070 IF 2008: 0.839	TMD	Pain, muscle disorders 6 months	TMJ, EO masticatory muscles & cervical (neck muscles)	Pain	40	1/39	20-68 GI: 40.15 GII: 46.6	NI	GI: LLLT GII: Placebo	At baseline & 4 weeks after treatment	Pain assessment & changes in TMD	VAS, CMI	SS between initial & final values of VAS & CMI ($p<0.05$) at intragroups. SS reduction in pain & TMJ symptoms in GI&II ($p<0.05$). Baseline & post-therapy, no SS in VAS & CM (p>0.05) of intergroup
Lassemi et al., 2008, Iran [63]	Journal of Oral Laser Applications IF 2019: (discontinued)	TMD	Pain, clicking	TMJ	Pain, clicking	48	24/24	>18	NI	GI: Experimental (LLLT) GII: Placebo	At baseline, immediately 2nd, 4th days, 6 & 12 months after treatment	Pain assessment , clicking in TMJ	VAS, clicking stethoscope	SS in PI reduction in GI than GII ($p<0.001$). SS in pain & clicking without recurrence up to 2-year, favouring GI ($p<0.001$).
Carrasco et al., 2008, Brazil [64]	Cranio: the Journal of Craniomandibul ar Practice IF 2019: 1.186 IF 2008: 0.846	TMD	Capsulitis, synovitis, retrodiscitis, displacement disc + reduction, TMJ pain +/ - tender muscles	TMJ, IO & EO masticatory cervical muscles	Pain, muscle tenderness	14	NI	> 18	NI	GI: LLLT GII: Placebo	Baseline, Ev1 (8th session), Ev2: (4th week) , Ev3: 30 days after 1st application	Pain assessment, masticatory efficiency	VAS, masticatory efficiency (CCM)	SS in TMJ pain reduction ($p<0.001$) in GI. No SS in masticatory behaviour in GI&II. In Ev2: improved symptoms & functionality throughout therapy.
Emshoff et al., 2008, Austria [65]	Oral Surgery, Oral Medicine, Oral Pathology, & Oral Radiology IF 2019: 1.791 IF 2008: 1.671	TMD	Unilateral TMJ pain. Pain onset of 2 years or less	TMJ	TMJ pain	52	GI: 4/22 GII: 6/20	18 -58 GI: 44.1±16.6 GII: 41.8±11.2	NI	GI: LLLT GII: sham LLLT	At baseline, 2nd, 4th & 8th weeks after treatment.	Pain assessment	VAS	At 8th week (intragroup): TMJ pain reduction during function, in G I& II (p=0.000), but differences were not highly evident ($p>0.05$) in intergroup
Carrasco et al., 2009, Brazil [66]	Cranio: the Journal of Craniomandibul ar Practice IF 2019: 1.186 IF 2010: 1.015	TMD	MPS with combination of regional & referred pain. MM restriction, pain in ant. masseter & ant. temporalis	Ant. of masseter & temporalis muscles	Pain & MM restriction	60	NI	>18	NI	GI-LLLT (25 J/cm^2), GII-LLLT (60 J/cm^2), GIII-LLLT (100 J/cm^2), GIV-LLLT Placebo (25 J/cm^2),	Ev1: Baseline Ev2: immediately after 4th application. Ev3: immediately after 8th application.	Pain assessment	VAS	A significant decrease in pain levels over time ($p<0.001$), in all groups, starting from Ev3. Placebo was as effective as LLLT in reducing PI ($p<0.05$). No SS between GI & III ($p>0.05$).

										GV-LLLT Placebo (60 J/cm^2), GVI-LLLT Placebo (100 J/cm^2)	Ev4: 15 days & 1-month after last session (Ev5)			
Shirani et al., 2009, Iran [67]	Lasers in Medical Sciences IF 2019: 2.342 IF 2009: 2.598	TMD	Myofascial pain 1month	Masticatory muscles, lateral & post. of TMJ	Myofascial pain	16	4/12	16-37 (23.8)	NI	GI: Laser GII: Placebo control	At baseline, immediately,1 week & 3 weeks after 1st therapy	Pain assessment	VAS	GI was more effective in PI reduction than GII (*p*=0.031).
Venezian et al., 2010, Brazil [68]	Cranio: the Journal of Craniomandibular Practice IF 2019: 1.186 IF 2010: 1.015	TMD	Myofascial pain	Bilateral ant. temporalis & masseter muscles (upper, medium & lower)	Myofascial pain	48	5/43	18-60 (41.58)	NI	GI-LLLT: 25 J/cm^2, GII-LLLT Placebo: 25 J/cm^2, GIII-LLLT: 60 J/cm^2 GIV-LLLT placebo: 60 J/cm^2	VAS: baseline, immediately after therapy (8th laser application & 30 days after treatment. EMG: pre & after 8th application (1 month).	Pain assessment, MVC, MVC with MHI	VAS, Surface EMG [MVC, MVC with MHI]	SS difference in masseter muscles tenderness in GI than GIV (*p*<0.05), but no SS differences in other masticatory muscles (intra & inter groups comparison) (p>0.05). SS (*p*<0.05) reduction in PI level in all palpated muscles of all GI,II,III
Oz et al., 2010, Turkey [69]	The Journal of Craniofacial Surgery IF 2019: 0.938 IF 2010: 0.873	myofascial pain	Orofacial pain 6 months	TMJ & EO masticatory muscles	Orofacial pain	40	SG: 3/17 CG: 3/17	G1: 31.25 ±8.23 CG: 34.52 ±12.82	NI	G1:: LLLT CG: Occlusal splints	Baseline- 30 mins before 1st session (week 1) & 30 mins after last session (week 5).	Mandibular mobility Masticatory muscles tenderness, pain assessment	Functional examination (RDC/TMD) , PPT (dial algometer), VAS	SS improvements in vertical movements of the muscles, tenderness on palpation (PPT&VAS) (*p*<0.01) in intragroup comparison, but not in intergroup comparison (*p*>0.01). LLLT is as effective as occlusal splint in pain release and MM improvement in MP.
Marini et al., 2010, Italy [70]	Clinical Journal of Pain IF 2019: 3.171 IF 2010: 3.900	TMD	Disc displacement without reduction or osteoarthritis	TMJ	NI	99 GI: 39 GII: 30 GIII: 30	25/74	15-55 GI:41.93 ±11.51 GII:36.23 ±11.30 GIII: 35.90 ±6.84	NI	GI: super-pulsed LLLT (SLLLT) GII: Drug therapy (Ibuprofen 800 mg BD, 10 days) GIII: sham SLLLT	Pain assessment: baseline, 2nd, 5th, 10th, 15th day & 1 month after therapy. MMO: baseline,15th day & 1 month after therapy. MRI: baseline & 1 month after therapy.	Pain assessment, MMO, MM TMJ soft tissue evaluation	VAS, MMO & jaw movements (millimetre ruler), MRI (TMJ soft tissue evaluation)	Inter group comparison revealed SS reduction in VAS scores, MMO & TMJ soft tissue evaluation for GI, compared to GII&III (*p*<0.001) at all corresponding follow-up time-points
Rohlig et al., 2011, Turkey [71]	Turkish Journal Physical Medicine and Rehabilitation IF 2019: 0.190 IF 2010: 0.242	Muscul.ar origin	Orofacial pain lasting for more than 6 months	TMJ, EO masticatory muscles & cervical (neck muscles)	Orofacial pain	40	GI: 16/24 GII: 8/12	43.7±1.8 GI: 22-59 (42.2±3.4) GII: 28-56 (42.8±2.1)	NI	GI: LLLT GII: Placebo	Baseline [30mins before 1st session (week 1)] & 30mins after last session (week 3).	MM, masticatory muscles, tenderness, pain assessment	Functional (RDC/TMD) , PPT (dial algometer), VAS	SS reduction in PPT, number of muscles with pain on palpation a& MM in GI (*p*<0.05) compared to GII.

Sattayut et al., 2012, United Kingdom [72]	Laser Therapy IF 2019: 1.05 IF 2012: NI	TMD	Unilateral myofascial pain	TMJ, EO & IO masticatory muscles	Myofascial pain	30	0/30	20-50	NI	G1:low energy LILT (CLILT) G2: modified high energy LILT (MLILT) G3: Placebo laser	Baseline (3 days prior to 1st LLLT session), 1st, 3rd, 5th & 8th day after 1st LLLT session.	PPT of TP in masticatory muscles, unassisted MOSP	PPT (dial algometer), MMO without pain (MOSP) [WBG], Jaw kinesiology, EMG & pain rating index (McGill), SSI	SS improvement in PPT (*p*=0.0001), EMG amplitude (*p*=0.022), myofascial pain (*p*=0.02) & TMJ arthralgia (*p*= 0.006) recorded from voluntary clenching (cEMG) in G2 compared to G3. At 2-4 weeks after LILT, 52% reduction in pain (SSI). No SS in any other parameters between G1,2,3 (*p*>0.05).
De Carli et al., 2012, Brazil [73]	Journal of Oral Rehabilitation IF 2021: 2.304 IF 2010: 2.761	TMD	Pain in TMJ region	TM J& EO masticatory muscles	TMJ pain	32	3/29	18-58 (32.4)	NI	GL: active laser & placebo piroxicam (11). GPi: placebo laser & piroxicam (10). G LPi: active laser & piroxicam (11)	At baseline & after 1st, 2nd, 3rd & 4th treatment sessions, 30 days post treatment	Subjective pain, mandibular mobility, joint & muscle related pain on palpation, MMO	VAS, MMO (millimetre ruler), palpation for pain assessment	SS improvement of VAS scores (*p*<0.05) in intragroup, but no SS intergroup differences. Reduction of pain in joint & muscles on palpation with Piroxicam(*p*<0.05) & lowest temporal pain (*p*=0.02) at 30-days follow-up. No difference between G LPi & single therapy (TMJ arthralgia)
da Silva et al., 2012, Brazil [74]	Cranio: the Journal of Craniomandibul -ar Practice IF 2019: 1.186 IF 2012: 1.422	TMD	TMJ pain 6 months	TMJ & EO masticatory muscles	TMJ pain	45	15/30	25-53 (39.7)	NI	GI: 52.5 J/cm² GII: 105 J/cm² GIII: Placebo	At baseline, after 1st,5th & 10th application & 5 weeks post treatment	Range of MM, MMO, pain assessment	VAS, MMO digital pachymeter	SS differences between the doses of GI&II at follow-up time points (*p*<0.05), favouring GII. LLLT increased MM & reduced the painful symptoms, compared GIII (*p*<0.05).
Panhoca et al., 2013, Brazil [75]	Lasers in Medical Sciences IF 2019: 2.342 IF 2013: 2.735	TMD	TMJ Pain during palpation	TMJ, masseter & temporalis muscles	Limited or painful MM & MO	30	8/22	18-40	NI	GI: red- LED 630±10 nm GII: IR- LED 850± 10 nm GIII: control, IR-red 780 nm	At baseline, 7th & 30th days after treatment.	Pain, MMO	NRS, jaw movement (RDR)	SS reduction in pain & an increase in MMO for all groups (*p*<0.05), but not between groups at baseline or at any period after treatment (*p*≥0.05).
Uemoto et al., 2013, Brazil [76]	Journal of Oral Science IF 2019: 1.2 IF 2013: 1.55	TMD	Myofascial TP in masseter muscle	Bilateral masseter muscles	Myofascial pain	21	0/21	20-52 years	NI	GI: LLLT GII: needle treatment	At baseline, after 4th & 8th day of treatment	Pain assessment, PPT, MMO, Masseter	VAS, PPT (DA), MMO (calliper), masseter	SS (p<0.05) reduction in PI (VAS) only in GI&II. SS increase in PPT in GII (*p*=0.0469) & GI at 4 J/cm²

										GIII: Placebo-laser (control)		muscle function	function (sEMG)	(*p*=0.0156). No SS in sEMG & MMO in all groups (*p*>0.05)
Ahrari et al., 2014, Iran [77]	Lasers in Medical Sciences IF 2019: 2.342 IF 2014: 2.700	TMD	Myogenic TMD, with/without limited MO	Bilateral TMJ, masseter (origin, body, insertion), temporalis (ant., middle, post.), MPM insertion	Limited MO	20	0/20	35.5	NI	GI: LLLT (experimental) GII: placebo (control)	T1: baseline T2: after 6 sessions of laser; T2: end treatment (2 weeks), T3: 4th week, T4: 1-month after last laser session	Level of pain MO	VAS, MMO (millimetre ruler)	SS increase in MO & reduction in pain in GI (*p*<0.05), compared to GII. Intergroup comparison, no SS in PI & MO measurement at any of evaluation time points (*p*>0.05).
Demirkol et al., 2014, Turkey [78]	Lasers in Medical Sciences IF 2019: 2.342 IF 2014: 2.700	TMD	MPDS (regional pain, reference pain pattern)	Ant. parts of masseter & temporal muscles	Myofascial pain	30	NI	>18	NI	GA: occlusal splint (n=10) GB: LLLT (n=10) GC: placebo (n=10)	At baseline & after treatment (10th day).	Pain assessment, Functional examination , pressure pain evaluation	Functional examination (RDC/TMD) VAS	SS reduction in PI in GA&B, compared to CG (*p*<0.05). No significant difference between GA&B after treatment (*p*>0.05).
Pereira et al., 2014, Brazil [79]	Cranio: the Journal of Craniomandibular Practice IF 2019: 1.186 IF 2014: 1.167	TMD	TP within pain score >5 on palpation, according NRS	Post. ligament & lateral pole TMJ; EO temporalis, masseter, post. mandibular & submand.; suboccipital, SCM, trapezius IO LPM, temporal tendon,	Pain	19	4/15	21-55	NI	G1: red-laser (660 nm) G2: IR-laser (795 nm)	At baseline, 24 hrs & 30th , 90th & 180th days after treatment	Pain assessment, QoL	NRS, QoL (OHIP-14)	SS in results of G1&2 (*p*<0.001), improvement in 24 hr-180th days in G1&2. SS difference between them at 180th days, favouring IR-laser (*p*=0.039).
Maia et al., 2014, Brazil [80]	Lasers in Medical Sciences IF 2019: 2.342 IF 2014: 2.700	TMD	Myofascial pain	EO masticatory muscles	Myofascial pain	21 GI:12 GII:9	2/19	27.76± 10.44	NI	GI: LLLT GII: Placebo	MP and PPT: at baseline, after last laser application & after 1 month VAS: at baseline, after each treatment & after 1 month	Masticatory performance (MP), PPT, pain assessment	MP [analysis of GMD of chewed particles by OTM], PPT (pressure algometer), pain assessment (VAS)	SS reduction GMD of crushed particles (*p*<0.01) & an increase in PPT (*p*<0.05) in GI compared to baseline & end-treatment values. A decrease in PI at end-treatment in GI: *p*<0.001; GII: *p*<0.01). At 30 days post treatment, a continuous reduction only in GI (*p*<0.001)
Sancakli et al 2015, Turkey [81]	BMC Oral Health IF 2019: 2.292 IF 2015: 1.652	TMD	MPDS	EO masticatory muscles	Myofascial pain	30	9/21	39.2	NI	G I: LLLT (greatest TP) GII II: LLLT of at pre-established TP of muscles GIII: Placebo	At baseline (30 min before 1st laser application & 30 min after the last laser application session (after 1 month).	MM, masticatory muscles tenderness, pain assessment	Functional examination (RDC/TMD) , PPT assessment (Dial algometer), VAS	SS in PPT, pain on palpation, MM range in both laser groups (*p*<0.05). SS in all measured values in GI than GII (*p*<0.05). No SS in any measured values in GIII (*p*>0.05).

De Oliveira et al., 2017, Brazil [82]	Cranio: the Journal of Craniomandibular and Sleep Practice<br>IF 2019: 1.186<br>IF 2017: 1.000	TMD	Myofascial pain	Post. ligament & lateral pole of TMJ; EO muscles (temporalis, masseter, post. mandibular & submand.); Suboccipital, SCM, trapezius. IO LPM, temporal tendon.	Myofascial pain	19	4/15	21-55<br>35	NI	G1: red-laser (660 nm)<br>G2: IR-laser (790 nm)	At baseline, 24 hrs, 30th , 90th & 180th days after treatment	Pain assessment, TMJ symptoms in muscles & ligaments	Pain assessment, pain survival time, recurrence of pain (KMM).	No SS between groups over follow-up (*p*>0.05). Both lasers were effective in reducing TMD symptoms & a low survival rate at 180th days (*p*=0.039). Grinding teeth (*p*<0.001) & headache (*p*=0.001) were associated with recurrent pain.
Costa et al., 2017, Brazil [83]	Brazilian Oral Research<br>IF 2019: 1.903<br>IF 2017: 1.223	TMD	Myalgia of temporalis & masseter muscles	Temporalis & masseter muscles	Myalgia	60	90% Females	38.8 (±14.2)<br>(18 – 76)<br>M:45.8<br>F: 38.9	NI	GA: Placebo<br>GB: LLLT	Ev1: at baseline<br>Ev2: end-treatment (single session).	Referred pain elicited by palpation, MMO	Muscle palpation (Dial algometer), VAS, MMO (digital calliper)	No SS intergroup differences in range for active or passive MO (*p*≥0.05). SS in total pain of right masseter muscle (*p*=0.001) & total PI (*p*=0.005). In EV2, SS differences in PI on palpation between GA&B for all masticatory muscles (*p*<0.05).
Seifi et al., 2017, Iran [84]	Journal of Lasers in Medical Sciences<br>IF 2019: 0.44<br>IF 2017: NI	TMD	Head & neck pain, tenderness on palpation, especially around ears & during function, limited MO	TMJ & masticatory muscles, including masseter, temporalis, LPM, MPM	Head & neck pain, tenderness on palpation, especially around ears & during function, limited MO	40	NI	18-50	NI	G1:TENS<br>G2: LLLT<br>G3:sham-TENS,<br>G3: sham-LLLT.	T1: baseline- T2-T5: after each session<br>T6:1-month post treatment	TMJ pain & tenderness, masticatory muscles, mandibular mobility	VAS, MMO (millimetre ruler)	A decrease in pain (*p*=0.000), tenderness (*p*=0.000) & an increase in MO (*p*=0.002) greater in G1&2 than G3&4.<br>At 1-month follow-up, SS reduction in pain & tenderness in G1&2 (*p*=0.000).<br>No SS between G1&2 & G3&4 in MMO at 1-month (*p*=0.692). Placebo improved symptoms with less important effects
Shobha et al., 2017, India [85]	Indian Journal of Dental Research<br>IF 2019: NI<br>IF 2017: NI	TMD	TMJ pain, joint noises, and muscle pain	TMJ & EO masticatory muscles	Pain, joint noises	40	G1: 3/17<br>G2: 6/14	18-40<br>G1: 30.85 ±6.31<br>G2: 27.55 ±4.58	NI	G1: LLLT<br>G2: Placebo	Pain: at baseline , end-treatment (4 weeks) & 30 days after treatment<br>MO: at baseline, end- treatment	Pain during function & at rest, mandibular mobility, TMJ clicking	VAS, MMO (millimetre ruler)	SS increase in MO in G1 (*p*=0.006) & G2 (*p*=0.021), No SS between groups pre- (*p*=0.247) & after therapy (*p*=0.330). SS reduction in clicking in G1 (*p*=0.000) & G2 (*p*=0.001). No SS between groups (*p*=0.752).
Rezazadeh et al.,	Journal of Dentistry (Shiraz University of	Drug-resistant TMD	Pain	Deep & superficial masseter, post., ant. & insertion of	Pain	45<br>G1: 19<br>G2: 15	G1: 5/14<br>G2: 4/11	G1: 31.87<br>G2: 30.79	NI	G1: TENS<br>G2: LLLT	Pain: at baseline & after each session (8), after	PI, clinical TMD evaluation	VAS, TMD evaluation	SS reduction in Helkimo index in G1&2 pre- & post-treatment (*p*<0.001);

2017, Iran [86]	Medical Sciences) IF 2019: NI IF 2017: NI			temporal, LPM, MPM ant. digastric, post. digastric & SCM							4th, 8th & 16th weeks post therapy. Clinical evaluation: at baseline & after treatment		(Helkimo index) (CBP)	but no SS between G1&2 (p=0.17). At 2nd session of treatment, SS reduction in PI (*p*<0.0001) in G1, whereas at 3rd session for G2 (*P*=0.007).
Varma et al., 2018, UAE [87]	World Journal of Dentistry IF 2019: 0.123 IF 2017: NI	TMD	Pain, clicking, MM limitation	TMJ, masseter, temporalis, & trapezius muscles	Pain, clicking, MM limitation	24 GI: 16 GII: 8	2/22	18-50	NI	GI: LLLT GII: Placebo laser	At baseline, immediately after each session (8) & 1-month after end-treatment	PI, clinical TMD evaluation	Wong & Baker pain scales	SS reduction in PI (*p*<0.0001) in GI compared to GII (*p*=1.000) pre- & after treatment. At 1-month post-treatment, SS reduction in PI (*p*=0.000) of GI&II.
Borges et al., 2018, Brazil [88]	Lasers in Medical Sciences IF 2019: 2.342 IF 2018: 2.263	TMD	Pain	Unilateral or bilateral TMJ	Pain	44	G1: 0/11 G2: 1/10 G3: 2/9 CG: 1/10	G1: 35.82 ±13.77 G2: 27.73 ±9.75 G3: 34.82 ±15.28 CG: 29.45 ±12.45	G1: 10 W/1B G2: 11 W/0B G3: 11 W/0B CG: 10 W/1B	G1: LLLT-8 J/cm^2 G2: LLLT-60 J/cm^2 G3: LLLT-105 J/cm^2 CG- placebo	At baseline & after 10th session (Approx. 3 weeks)	Pain assessment, symptom severity, joint mobility, MO	VAS, TMD evaluation Anamnestic TMJ mobility	SS increase in MO in G1 (*p*<0.05). SS decrease in PI in all groups (*p*<0.05). An effective reduction in TMJ pain & other symptoms at all tested doses of 830nm laser-PBM, but 8 J/cm^2 most effective in MMO & MP (*p*<0.05).
Brochado et al., 2018, Brazil [89]	Brazilian Oral Research IF 2019: 1.903 IF 2018:1.544	Myogenic & atherogen-ic	Pain in TMJ & limited MO	TMJ, IO & EO masticatory muscles	Pain in TMJ & limited MO	41	G1:LLLT (0/14) G2: MT (2/12) G3: combined (CT) (1/13)	G 1: LLLT (45.7 ± 15.7) G2 :MT (41.2 ± 20.3) G3: CT (46.5 ± 14.4)	NI	G1: LLLT G2: MT G3: combined (CT)	At baseline,7th , 14th , 21st , 28th , 60th , 90th days	PI, MM, psychosocia-l aspects & anxiety	VAS, functional examination (RDC/TMD) , anxiety assessment (BAI)	SS reduction in PI & improvement in MM during treatment & at follow-up (*p*<0.001) in G1,2,3, but from psychosocial aspects, no effect in PI modification (*p*>0.05). SS reduction in depression in G1&3 (*p*≤0.05). SS reduction in physical symptoms +/- pain & improvement in jaw disabilities (*p*≤0.05) in G1,2&3. MT: improvement in 5 functions, 2 in G1 & 1 in G2 (*p*<0.001). SS reduction in anxiety in G1,2,3 (*p*≤0.05). No more benefits from G3 compared to G1&2 alone.
Rodrigues et al., 2018, Brazil [90]	Cranio: the Journal of Craniomandibul	TMD	Muscle pain in the face, joint	TMJ, IO & EO masticatory muscles	Muscle pain	78	0/78	18–60 (31.94 ± 9.57)	NI	G1: Active laser (30) G2: Placebo laser (29)	At baseline, end-treatment (4th week) & 30 days post treatment	OMC, TMD severity, PI, PPT	Masticatory test, OMES, VAS, PPT	Reduction of PI during chewing & better recovery levels during rest period (*p*>0.05) in

	ar and Sleep Practice IF 2019: 1.186 IF 2017: 1.047		dysfunction 6 months							G3: Control (19)			(dial algometer)	G1&2, without differences in OMC between groups. Means for OMES were similar between G1,2&3 (p<0.05).
Peimani et al., 2018, Iran [91]	Journal of Oral Health and Dental Sciences IF 2019: NI IF 2018: NI	TMD	Dysfunction of masticatory muscles, restrictive jaw movements, bruxism or clenching, clicking sound & sensitivity to palpation at TMJ	TMJ, masticatory muscles (EO)	Masticatory muscles dysfunction, restrictive jaw movements, bruxism or clenching, clicking sound & TMJ sensitivity	72	G1: 12/24 G2:14/22	20-45 G1:29.06± 6.85 G2:28.72± 9.47	NI	G1: Drug therapy (Naproxen 500mg & diazepam, BD,10 days) G2: LLLT	At baseline, immediately, 1st, 2nd & 4th weeks after the treatment.	Pain assessment, clicking & TP tenderness	VAS, AROM	A reduction in pain, clicking& tenderness in G1&2. SS reduction in PI values on VAS in G1 immediately after treatment (p<0.01). SS increase in AROM index, in G2 (p<0.01). laser & drug therapies were effective, but significant decrease in recovery process in G2.
Nadershah et al., 2019, Saudi Arabia [92]	Journal of Maxillofacial and Oral Surgery IF 2019: NI	Myofascial pain	Unilateral TMJ & masticatory muscles pain during function	TMJ, masticatory muscles (EO)	TMJ & muscle pain during function	212 GI: 108 CG: 94	PBMT: 49/59 Control: 43/51	GI: LLLT 19-58 (34.3 ±10.5) CG: 18-58 (33.3 ±10.7)	NI	GI: LLLT CG: Sham laser	At baseline & up to 10 days, every 48hr	Pain assessment	VAS	Statistically significant reduction in PI in GI compared to CG (p=0.01).
Magri et al., 2019, Brazil [93]	Cranio: the Journal of Craniomandibular and Sleep Practice IF 2019: 1.186	Myofascial pain	Pain in facial region At least 3 months	TMJ, EO masticatory muscles	Facial pain during function	41	0/41	31.7±5.2	NI	GI: Laser: 20 GII: Placebo: 21	At baseline, immediately after the last laser session (T8), 4th weeks & 6th,12th months after treatment	Pain on palpation, referred pain MM	VAS, functional examination (RDC/TMD)	At T8 & 6 months, reduction in PI (p<0.05) in GI& II. At 1-year, GI&II showed no difference in PI (p>0.05). Active LLLT was more effective in reducing pain on palpation (p=0.001) & referred pain (p=0.04) in TMJs regions
Al-Quisi et al., 2019, Iraq [94]	Pain Research and Treatment IF 2019: 2.153	TMD	Myofascial pain	TMJ, IO & EO masticatory muscles	Myofascial pain	50	10/40 GA: 7/18 GB: 3/22	19-24 GA: 21.8 GB: 19.5	NI	GA: LED-LLLT GB: Placebo	At baseline & each visit (once a week for 4 weeks)	Pain assessment	VAS	The changes in PI values & no. of tender muscles in both groups were highly significant, only GB less but with no significant differences (p>0.05).
Herpich et al., 2019, Brazil [95]	Lasers in Medical Sciences IF 2019: 2.342	TMD	Myogenous TMD	LPM	Moderate to severe pain on palpation	30	0/30	GA: 25.44 ±5.76 GB: 26.55 ±4.6	NI	GA: LLLT GA: Sham LLLT (CG)	At baseline, end 1st session, then 24 hr & 48 hr after that & after 6 sessions.	Pain assessment, Range of MM, TMJ functioning	VAS, MMO (digital calliper), TMJ functioning (functional scale)	SS improvement in PI (p≤0.01) & functioning (p≤0.04) in GA than GB, but no SS difference in range of MM (p>0.05)

Khairnar et al., 2019, India [96]	Journal of Dental Anesthesia and Pain Medicine IF 2019: NI	TMD	TMD-related pain > 3 months	TMJ	TMD-related pain	42	20/22	25-45 (37±2.13)	NI	GA: LLLT GB: Ultrasound therapy	At baseline & 30 days after treatment	Pain assessment, MM	VAS, MMO (Flexible millimetre ruler)	SS in the mean VAS & mean MO values, favouring GA (p<0.05).
Sobral et al., 2020, Brazil [97]	Research Society and Development IF 2019: NI	TMD	Myofascial pain, disc displacement with reduction	EO masticatory muscles	Myofascial pain	23 (GA:12, GB:11)	2/21	22.91	NI	GA: LLLT GB: Occlusal splints	At baseline & 30 days after treatment	Pain assessment, QoL, cost-effective-ness	VAS, functional examination (RDC/TMD) , EuroQol-5D	A decrease in PI on VAS, in GA&B, before & after 1 month of therapy (p<0.05) with no difference between groups. Positive impact on QoL in GA&B with higher value in GA (p<0.05)
Maracci et al., 2020, Brazil [98]	Cranio: the Journal of Craniomandibular and Sleep Practice IF 2019: 1.186	TMD	Myofascial pain (with or without MM limitation)	TMJ, EO masticatory muscles	Myofascial pain +/- MM limitation	31	73.3% female	18-60 (mean-33.27)	NI	G1: occlusal splint G2: LLLT G3: LLLT (sham)	At baseline &1 month after treatment.	Pain on palpation, Oral Health related QoL	Functional examination (RDC/TMD) , QoL (OHIP)	An improvement in PI (p=0.014) & myofascial pain diagnosis in G1 (p=0.008) compared to G2&3. SS improvement in OHR-QoL in G1&2 (p=0.005) than G3.
Chellappa et al., 2020, India [99]	Indian Journal of Dental Research IF 2019: NI	TMD	Persistent, recurrent, or chronic TMJ pain	TMJ	TMJ pain	60	NI	Above 18	NI	GA: LLLT GB: TENS	At baseline, after each laser session (6 sessions)	Pain assessment, MM	VAS, NRS Distress scale	Significantly higher reduction in PI & increase in MM in GA more than GB (p<0.01).
Monteiro et al., 2020, Brazil [100]	Photobiomodulation, Photomedicine, and Laser Surgery IF 2019: 1.918	Unilateral / bilateral TMD	Painful myofascial TMD	TMJ & EO & IO muscles mastication	Myofascial pain	42	10/32 GA: 5/15 GB: 5/17	27.4±9.71 GA:29.1 ±11 GB:25.6 ±8	NI	GA: LLLT GB: Placebo	At baseline & 1 month after treatment.	Non-assisted & perception pain MM, tenderness elicited by palpating the muscles	VAS, MMO (pachy-meter)	SS reduction in PI in GA compared to GB after treatment (p<0.001). SS increase in non-assisted painless MM in GA compared to GB (p=0.007).
