# Supplementary material for: Role of Photobiomodulation Therapy in Modulating Oxidative Stress in Temporomandibular Disorders. A Systematic Review and Meta-Analysis of Human Randomised Controlled Trials
Source: Antioxidants (Basel). 2021 Jun 25;10(7):1028. doi: 10.3390/antiox10071028 (PMC8300797; doi:10.3390/antiox10071028)
Supplement: Supplementary file 1 [file antioxidants-10-01028-s001.zip › supplementary-2021.7.1/Table S2 .pdf]

**Table S2.** Tabular representation of lasers/LEDs parameters utilised in the chosen eligible *in vivo* RCTs human studies related to temporomandibular dysfunction syndrome (TMD). The abbreviations in this table are listed in Supplementary File S2

| Study, year, origin & citation | Utilised Laser/ LED wavelength | Emission mode CW/ Gated/ Pulsed | Energy (J) | Power output (W) | Pulse width (PW) & frequency (F) | Use of Power meter | Method of applications (EO or IO) & number of trigger points (TP) | Contact /non-contact | Tip-tissue distance | Spot size/spot area/beam & fibre tip diameters | Irradiated/ illuminated area | Energy density [Fluence] (J/cm²) | Power Density (W/cm²) | Exposure time (min/sec) | No.& frequency of sessions | Treatment duration |
|---|---|---|---|---|---|---|---|---|---|---|---|---|---|---|---|---|
| Venancio et al., 2005, Brazil [57] | 780nm GaALAs diode laser | NI | NI | 30mW | NI | NI | Bilateral EO: 3 TMJ points (a) post. aspect of joint (mouth open) (b) ant. to condyle in sigmoid notch (mouth closed), into condylar neck & disc (insertion of LPM); (c) joint interface with mouth open | NI | NI | NI | NI | 6.3J/cm²/ point | NI | 10sec | 6 sessions (Twice a week) | 3 weeks |
| Çetiner et al., 2006, Turkey [58] | 830nm GaAlAs diode laser | NI | NI | NI | NI | NI | EO: 4 TP of the following: joint capsule (lateral, post., sup.), masseter (ant., inf., deep), temporal (ant., deep, middle, origin), MPM; LPM [Bilateral application] | Contact | NA | NI | NI | 7J/cm²/ point | NI | 162sec | 10 sessions daily (excluding weekends) | 2 weeks |
| Fikackova et al., 2007, Czech Republic [59] | 830nm GaAlAs diode laser | NI | NI | 400mW | NI | NI | EO: For myofascial pain: painful muscle spots:, for TMD arthralgia: front of tragus (mouth open), through external acoustic meatus (mouth closed); 2cm in front of tragus, under zygoma (mouth closed) (No. of TP or allocation unspecified) | Contact | NA | Probe aperture size: 0.2cm² | NI | GA: 10J/cm²/ point GB: 15J/ cm²/point GC: 0.1J/ cm²/point | NI | NI | 10 sessions (Interval time unspecified) | 4 weeks |
| Mazzetto et al., 2007, Brazil [60] | 780nm GaALAs diode laser | CW | NI | 70mW | NA | NI | EO: TMJ region, at 1 point: inside the external auditive duct towards retrodiscal region [Bilateral application] | Contact | NA | NI | NI | 89.7J/cm²/ point | NI | 10sec | 8 sessions (Twice a week) | 4 weeks |
| Frare et al., 2008, Brazil [61] | 904nm GaAs diode laser | CW | NI | 15mW | NA | Yes | EO: 5 TP: 4 in a shape of a cross in the pre-auricular region; & 1 in external auditory meatus [Bilateral application]. | Contact | NA | Beam area: 0.039cm² | NI | 7J/cm²/ point | 0.38mW/cm²/ point | 16 sec/point | 8 sessions (Twice a week) | 4 weeks |
| Da Cunha et al., 2008, Brazil [62] | 830nm GaAlAs diode laser | NI | 4J/point | 500mW | NI | NI | EO: painful areas (No. of TP or allocation unspecified) | Contact | NA | NI | NI | 100J/cm²/ point | NI | 20sec/site | 4 sessions (Once a week) | 4 weeks |

| | | | | | | | | | | | | | | | | |
|---|---|---|---|---|---|---|---|---|---|---|---|---|---|---|---|---|
| Lassemi et al., 2008, Iran [63] | 980nm diode laser | Puled | 2J/point (Total: 6J) 1.5J at the other sites of muscle pain | NI | F: 80 Hz PW: NI | NI | EO: (a) post. part of joint-mouth open ( post. articular branches of auriculotemporal nerve) (2 J for 1 min), (b) an area ant. to condyle in sigmoid notch-mouth closed for LPM insertion in condylar neck & meniscus (2 J for 1 min); (c) joint interface-mouth open (2 J for 1 min); TP in adjacent muscles (No. of TP unspecified). | Contact | NA | NI | NI | NI | NI | 1min | 2 sessions (48-h interval) | 4 days |
| Carrasco et al., 2008, Brazil [64] | 780nm GaAlAs diode laser | CW | NI | 70mW | NA | NI | EO: 5 points of the TMJ area: lateral, sup., ant., post. & posterior-inferior of condylar [Bilateral application]. | Contact | NA | NI | NI | 105J/$cm^2$/ point | NI | 60sec | 8 sessions (Twice a week) | 4 weeks |
| Emshoff et al., 2008, Austria [65] | HeNe 632.8nm diode laser | CW | NI | 30mW | NA | Yes | EO: centre of the upper joint space, 1 cm in front of the tragus (No. of TP or allocation unspecified) | Contact | NA | NI | NI | 1.5J/$cm^2$/ point | NI | 2 mins at both closed mouth & MMO position | 1 series of 20 sessions (2 to 3 times a week) | 8 weeks |
| Carrasco et al., 2009, Brazil [66] | 780nm GaAlAs diode laser | CW | NI | GI: 50mW GII: 60mW GIII: 70mW GIV: 50mW Placebo GV: 60mW Placebo GVI: 70mW Placebo | NA | NI | EO: (No. of TP or allocation unspecified) | Contact | NA | NI | NI | GI-LLLT: 25J/$cm^2$/ point GII- LLLT: 60J/$cm^2$/ point GIII- LLLT: 100J /$cm^2$/ point. GIV-LLLT Placebo: 25J/ $cm^2$/ point GV-LLLT Placebo: 60J /$cm^2$/ point GVI- LLLT, Placebo: 100J /$cm^2$/ point | NI | NI | 8 sessions (Twice a week) | 4 weeks |
| Shirani et al., 2009, Iran [67] | Two diode laser probes (660nm & 890nm) | 660 nm: CW 890 nm: Pulsed | 6-10 J/ point | 660nm: 17.3mW 890nm: 9.8W | F:1500 Hz PW: NI (890nm) | Yes | EO: tender TP of masseter. IO: MPT, LPM (No. of TP or allocation unspecified) | Contact | NA | Spot size: 0.6$cm^2$ Irradiated area: 1 $cm^2$ | NI | 660nm: 6.2J /$cm^2$/ point 890nm: 1J/$cm^2$/ point | NI | 660 nm: 6 min 890 nm:10 min | 6 sessions (Twice a week) | 3 weeks |
| Venezian et al., 2010, Brazil [68] | GaAlAs diode laser (780nm) | CW | NI | GI- 50mW | NA | NI | EO: 4 points (upper, middle & lower thirds of masseter (3 points) & ant. | Contact | NA | NI | NI | GI:25J/$cm^2$ /point | NI | GI: 20 secs | 8 sessions (Twice a week) | 4 weeks |

| | | | | | | | | | | | | | | | | |
|---|---|---|---|---|---|---|---|---|---|---|---|---|---|---|---|---|
| | | | | GII-50mW Placebo GIII-60mW GIV-60mW Placebo | | | region of temporalis (1 point) | | | | | GII: 25J/cm$^2$/ point, Placebo GIII: 60J/ cm$^2$/point GIV:60J/cm$^2$/ point, Placebo | | GII: 20 secs GIII: 40 secs GIV: 40 secs | | |
| Oz et al., 2010, Turkey [69] | Low-intensity semiconduc-tor diode laser:820nm | CW | NI | 300mW/ point | NA | NI | EO: 3 points of masseter (sup., middle, inf.), 1point of temporalis (ant.) on both sides | Non-contact | 2mm | Beam diameter: 6mm | NI | 3 J/cm$^2$/ point | NI | 10 secs/ point | 10 sessions (Twice a week) | 5 weeks |
| Marini et al., 2010, Italy [70] | 910nm GaAs diode | Super-pulsed | NI | Average power: 400mW Peak power: 45W | F:1-50 kHz PW: <200 ns | Yes | EO: in right and left TMJ (No. of trigger points or allocation not mentioned) [Bilateral application]. | Contact | NA | NI | NI | NI | NI | 3 steps therapy/ TMJ 120 kHz for 10 mins 2.18k Hz for 5 mins 3.16 kHz for 5 mins | 10 sessions (10 consecutive days ; 5 days/week) | 2 weeks |
| Rohlig et al., 2011, Turkey [71] | Low-intensity semiconduc-tor (820nm) diode laser | CW | NI | 300mW/ point | NA | NI | EO: 5 points 3 on masseter (sup., middle, inf.), 1 on temporalis (ant.) & 1 on sup. SCM. | Contact | NA | Beam diameter: 6mm | NI | 8J/cm$^2$/point | NI | 10 sec/point | 10 sessions (Every alternate day) | 3 weeks |
| Sattayut et al., 2012, United Kingdom [72] | 820nm GaAlAs diode laser | NI | G1: 4J/ point G2: 20J/ point | G1: 60mW G2: 300mW | NI | NI | EO: 6 points Post. TMJ, sigmoid notch, joint interface, 3 tender TP in masticatory muscles elicited by palpation [Unilateral application] | Contact | NA | NI | NI | G1: LLLT-21.4J/cm$^2$/ point G2: LLLT-107J/cm$^2$/ point | NI | NI | 3 sessions (Every alternate day) | 1 week |
| De Carli et al., 2012, Brazil [73] | 808nm GaAlAs diode laser | NI | 2.8 J/point Total: 56 J | 100mW/ point | NI | Yes | EO: 10 TP of TMJ & muscle: joint capsule (lateral, post., sup., ant., inf.), masseter (origin, insertion) & temporal (ant., middle, post.). [Bilateral application] | NI | NI | Spot size: 0.028cm$^2$ | NI | 100J/cm$^2$/ point | NI | 28 secs | 4 sessions (Twice a week) | 10 days |
| da Silva et al., 2012, Brazil [74] | 780nm GaAlAs diode laser | CW | NI | 70mW | F: NA PW: NA | NI | EO: 5 TP Condylar points (ant., sup., post., post.-inf.) & external auditory meatus), 3-masseter & 1-ant. temporalis [Bilateral application] | Contact | NA | Beam diameter: 5mm | NI | GI: 52.5J/ cm$^2$/point GII: 105J/cm$^2$/ point GIII: Placebo | NI | GI:30 sec GII:60 sec GIII:30 sec | 10 sessions (Twice a week) | 5 weeks |

| | | | | | | | | | | | | | | | | |
|---|---|---|---|---|---|---|---|---|---|---|---|---|---|---|---|---|
| Panhoca et al., 2013, Brazil [75] | GI: red-LED (630±10nm), GII: IR-LED (850± 10nm) GIII: Control positive, IR-laser (780nm) | NI | GI & GII: 9J/ point GIII:4.2J /point | GI and GII: 150mW GIII: 70mW | NI | Yes | EO: 5 TP: 3 points around TMJ, 1 point for temporalis & 1 point near the masseter [Bilateral application]. | Contact | NA | GIII: Spot size: 0.04cm$^2$ | GI &GII: Illuminati-on area: 0.5cm$^2$ | GI and GII: 18J/cm$^2$/ point GIII: 105J/cm$^2$/ point | GI & GII: 300mW/cm$^2$/ point GIII: 1.7W/ cm$^2$/ point | 60 sec/ point | 8 sessions (Twice a week) | 4 weeks |
| Uemoto et al., 2013, Brazil [76] | 795nm IR-diode laser | NI | NI | 80mW | NI | NI | EO: MTPs in masseter (No. of TP or allocation unspecified) [Bilateral application]. | Contact | NA | NI | NI | Right masseter: 4J/ cm$^2$/point Left masseter 8J/cm$^2$/point | NI | NI | 4 sessions 72 hr interval between 1&2 sessions; 48 hr interval between 2&3 session; 72 hr interval between 3&4 sessions | 8 days |
| Ahrari et al., 2014, Iran [77] | 810nm diode laser | Pulsed mode | 6J/ point | 50mW/ point | F: 1500 Hz PW: 1µs | Yes | EO: masseter (origin, body, insertion); temporalis (ant., middle, post.) & MPM insertion (No. of TP unspecified) [Bilateral application]. | Contact | NA | Spot size: 1.76cm$^2$ | NI | 3.4J/cm$^2$/ point | NI | 120 sec/ point | 12 sessions (3 times a week) | 4 weeks |
| Demirkol et al., 2014, Turkey [78] | Nd:YAG, 1064nm | Pulsed | NI | 250mW/point | F: NA PW: NA | NI | EO: TP of masticatory muscles (No. TP or allocation unspecified) | Non-contact | 1cm$^2$ | NI | NI | 8J/cm$^2$/point | NI | 20 sec/ point | 10 sessions (Once a day) | 10 days |
| Pereira et al., 2014, Brazil [79] | 660nm (red laser) & 795nm (IR- laser). | NI | NI | NI | NI | NI | EO: TMJ (post. ligament & lateral pole); EO muscles (temporalis, masseter, post. mandibular & submand. regions; IO muscles (LPM & temporal tendon). Cervical muscles (suboccipital, SCM & trapezius). (No. of TP unspecified) | NI | NI | NI | NI | Muscle: 8J/cm$^2$/point TMJ: 4J/cm$^2$/point | NI | NI | 3 sessions (48 hr interval time) | 1 week |

| | | | | | | | | | | | | | | | | |
|---|---|---|---|---|---|---|---|---|---|---|---|---|---|---|---|---|
| Maia et al., 2014, Brazil [80] | 808nm GaAlAs diode laser | CW | Total: 1.9J | 100mW | NA | NI | EO: 5 TP: ant. temporalis & masseter, 4 forming a cross & 1 central point; distance between each point: 1cm [Bilateral application] | NI | NI | Spot area: 0.028$cm^2$ | NI | 70J/$cm^2$/point | NI | 19 sec/ point | 8 sessions (Twice a week) | 4 weeks |
| Sancakli et al., 2015, Turkey [81] | 820nm diode laser | CW | NI | 300mW/point | NA | NI | EO (Bilateral) LGI: LLLT Greatest painful points on masseter and/or temporalis (No. of TP or allocation unspecified). LGII: 6 TP: 3 on masseter (sup., middle, and inf.) & 3 on temporalis (ant., middle, post.) | Non-contact | 2mm | Beam diameter: 6mm | NI | 3J/$cm^2$/point | NI | 10 secs/ point | 12 sessions (3 times a week) | 4 weeks |
| De Oliveira et al., 2017, Brazil [82] | GaAlAs diode laser Red-laser: 660nm IR-laser: 790nm | NI | NI | Muscle points: 120 mW/ point Joints pain: 120mW/ point | F: NI PW: NI | NI | EO: TMJ (post. ligament & lateral pole); EO muscles (Temporalis, masseter, post. mandibular & submand. regions); IO muscle (LPM & temporal tendon) (No. of TP or allocation unspecified) | NI | NI | Spot size: 0.031$cm^2$ | NI | Muscle points: 8J/$cm^2$/point Joints with sensitivity: 4J/$cm^2$/ point | NI | Muscle TP: 1.06 sec/ point Sensitive joints: 0.33 sec/ point | 3 sessions (At an interval of 48 hrs) | 1 week |
| Costa et al., 2017, Brazil [83] | IR-laser (830nm) | NI | 2.8 J/ point Total: 14J | 100mW/ point | F: NI PW: NI | Yes | EO: 5 TP temporalis (ant., medium, post.) superficial masseter (sup., inf.)[Bilateral applications] | NI | NI | Spot size: 0.028$cm^2$ | NI | 100J/$cm^2$/ point | NI | 28 secs /point | 1 session | 1 day |
| Seifi et al., 2017, Iran [84] | Diode Laser (810nm) | CW | NI | 0.5W | NA | NI | (No. of TP or allocation unspecified) | NI | NI | Spot size- 5mm | NI | NI | NI | 60 sec/ point | 4.5 sessions/ week | 1 week |
| Shobha et al., 2017, India [85] | GaAlAs 810nm diode laser | CW | NI | 100mW | NA | NI | EO: at the centre of upper joint space, 1cm in front of tragus & at TP (No. of TP or allocation unspecified) | Non-contact | NI | Tip diameter: 300μ | NI | 6J/$cm^2$/point | NI | 60 sec/ session | 8 sessions (2–3 times a week) | Approx. 4 weeks |
| Rezazadeh et al., 2017, Iran [86] | GaAlAs 980nm diode laser | NI | NI | 200mW | F: NI PW: NI | NI | EO: 3 regions of both sides including post. & ant. aspects of TMJ, as well TP (No. of TP or allocation unspecified) | NI | NI | NI | NI | 5J/$cm^2$/point | NI | 2.5 mins | 8 sessions (Time interval was not mentioned) | 2 weeks |
| Varma et al., 2018, UAE [87] | 940nm diode laser | CW | NI | 6W | NA | NI | EO: Painful muscular areas over TMJ, masseter, temporalis, trapezius. (No. of TP or allocation unspecified) | Non-contact | 1cm up to 3cm | NI | NI | NI | NI | 300 sec | 8 sessions (Twice a week) | 4 weeks |

| | | | | | | | | | | | | | | | | |
|---|---|---|---|---|---|---|---|---|---|---|---|---|---|---|---|---|
| Borges et al., 2018, Brazil [88] | AlGaAs diode laser (830nm) | CW | G1: 7.68 J<br>G2:57.6 J<br>G3: 101.12J | 30mW | NA | Yes | EO: 8 TP (4/side) in TMJ: preauricular region & external acoustic meatus | Contact | NA | Spot size: 0.11600$cm^2$ | NI | G1: 8 J/$cm^2$/ point (Total: 64J/$cm^2$),<br>G2: 60J/$cm^2$/ point (Total: 480J/$cm^2$),<br>G3: 105J/$cm^2$/point (Total 840J/$cm^2$) | 2.59 W/ $cm^2$/ point | G1: 32sec/ point; 266 sec/ session; 2586sec/ 10 sessions<br>G2: 240 sec/point; 1920sec/ session; 19200 sec /10 sessions<br>G3: 420 sec /point; 3360 sec /session; 33600 sec/ 10 sessions | 10 sessions (3 times a week) | 3 weeks |
| Brochado et al., 2018, Brazil [89] | GaAlAs 808nm diode laser | CW | 4 J/ point;<br>48 J/ point or 576 J /side | 100mW/ point | NA | Yes | EO: 12 points/side:<br>5 in TMJ (sup., ant., lateral, post., postero-inferior to condyle) & 7-temporalis (ant., middle, & post.), masseter (upper, middle, lower), & insertion of MPM | Contact | NA | Spot size: 0.03$cm^2$ | NI | 13.3J/$cm^2$ /point<br>Radiant exposure: 133J/$cm^2$ | 3.33 W/ $cm^2$/ point | 40sec/ point | 12 sessions (3 times a week) | 4 weeks |
| Rodrigues et al., 2018, Brazil [90] | GaAlAs 780nm diode laser | CW | NI | Site 1: (60mW)<br>Site 2: (60mW) | NA | NI | EO: Site 1: superficial masseter (upper, middle, lower), Temporalis (ant., middle, post. Site 2: lateral pole of TMJ (5 TP related to lateral pole of mandible head: lateral, sup., ant., post., inf.) [Bilateral application]. | Contact | NA | NI | NI | Site 1: 30J/$cm^2$/ point<br>Site 2: 75J/$cm^2$/ point | NI | Site 1: 20 sec<br>Site 2: 50 sec | 8 sessions (Twice a week) | 4 weeks |
| Peimani et al., 2018, Iran [91] | 808nm diode laser | NI | 4J/ point | 50mW | F: NI<br>PW: NI | NI | EO: 3 TP over TMJ/ session (allocation of TP unspecified) | Contact | NA | Spot size: 0.028$cm^2$ | NI | 144J/$cm^2$/ point | 1.78 W/ $cm^2$/ point | 10 sec/ point | 8 sessions (Twice a week) | 4 weeks |
| Nadershah et al., 2019, Saudi Arabia [92] | 940nm diode laser | CW | 300J/ Rx Total dose: 1500J | 7W | NA | NI | EO: 5 TP at the temporal (centre of temporalis), zygomatic (origin of masseter), angle of mandible (masseter | Non-contact | 2cm | Spot size: 2.8$cm^2$ | NI | 300J/$cm^2$/ point | NI | 2 min/ application (24 sec/ point) | 5 sessions (Every 48 h) | 10 days |

| | | | | | | | | | | | | | | | | |
|---|---|---|---|---|---|---|---|---|---|---|---|---|---|---|---|---|
| | | | | | | | insertion), pre-auricular & mastoid areas [Unilateral application]. | | | | | | | | | |
| Magri et al., 2019, Brazil [93] | GaAlAs diode laser 780nm | CW | NI | Masseter & ant. temporal (20mW); TMJ (30mW) | NA | NI | EO: masseter & anterior temporal; TMJ Distance between points: 1 cm (No. of TP or allocation unspecified) | Contact | NA | NI | NI | Masseter & ant. temporal: 5J/cm²/ point TMJ: 7.5J/ cm²/point | NI | 10 sec/ point | 8 sessions (Twice a week) | 4 weeks |
| Al-Quisi et al., 2019, Iraq [94] | Red LED 660nm | NI | Total: 1.6J | 1.6W | F:NI PW:NI | NI | EO: TMJ, LPM & tender TP on masseter & temporalis (No. of TP or unspecified) | NI | NI | NI | NI | NI | NI | 3 mins/ point | 4 sessions (Once a week) | 4 weeks |
| Herpich et al., 2019, Brazil [95] | Cluster of 9 diodes: one super-pulsed laser diode (905nm), 4 red LED diodes (640nm) & 4 IR-LED diodes (875nm LED). | Laser 905nm: super-pulsed LED (640nm & 875nm): pulsed | Laser total energy 0.27J 640nm LED: 4.5J/ emitter, total: 18 J 875nm LED: 5.25J/ emitter, total: 21 J Total energy: 39.27 J | 905nm diode laser: 0.9 mW [mean optic output (MOO)] 640nm LED: 15mW (MOO) 875nm LED: 17.5mW (MOO) | F: 1000Hz (905nm diode laser) F:2 Hz (640 nm LED) F: 16 Hz (875 nm LED) PW: NI | Yes | IO: Region of pterygoid muscles (No. of TP or allocation unspecified) [Bilateral application]. | Contact | NA | Laser tip size: 0.4cm² 640 nm-LED: 0.9cm² 875 nm-LED: 0.9cm² Aperture size: 4cm² but an adapter with an aperture of 0.394cm² for IO | NI | 99.67J/cm²/ point | NI | Laser: 300 sec 640 nm LED: 300 sec 875 nm LED: 300 sec | 6 sessions (unspecified time interval) | NI |
| Khairnar et al., 2019, India [96] | 660nm diode laser | NI | 2.2 J/min | 60mW | NI | NI | EO: TMJ region- at centre of upper joint space, 1 cm in front of tragus (No. of TP or allocation unspecified) | Contact | NA | NI | NI | NI | NI | 3 mins | 15 sessions (an alternate day) | Approx. 4 weeks |
| Sobral et al., 2020, Brazil [97] | 808nm±10 nm InGaAlP diode laser | CW | 6 J/ point | 100mW | NA | NI | EO: Total: 8 TP: 3 on masseter (upper, middle, lower) & 1 point on ant. temporalis [Bilateral application] | Contact | NA | Beam diameter with spacer: 0.354cm Beam area: 0.0984cm² | Irradiated area: 0.7872cm²/point | 61J/cm²/point | 1016mW/ cm²/point | 60 secs/ point | 12 sessions (Twice a week) | 6 weeks |
| Maracci et al., 2020, Brazil [98] | GaAlAs 808nm diode laser | CW | NI | 100mW | NA | NI | EO: 24 TP; 1 cm distance between two sites on TMJ & associated musculature | Contact | NA | NI | NI | 80J/cm²/point | NI | 22 sec/ application | 2 sessions (48 hr interval) | 3 days |

| | | | | | | | | | | | | | | | | |
|---|---|---|---|---|---|---|---|---|---|---|---|---|---|---|---|---|
| Chellappa et al., 2020, India [99] | 672nm diode laser | NI | 3J/per site | 50mW | NI | NI | EO: 4 sites (masseter, temporalis, condylar regions & intra-auricular portion) | Contact | NA | NI | NI | NI | NI | 120 sec/ point | 6 sessions (Twice a week) | 3 weeks |
| Monteiro et al., 2020, Brazil [100] | 635±10nm diode laser | CW | 16J/ point & Total: 128J | 200mW | NA | NI | EO: depending on painful points (diagnostic questionnaire) at palpation (average of 4 TP/ side) (No. of TP or allocation unspecified) [Bilateral application]. | Contact | NA | Beam diameter: 8mm Beam spot size $0.5cm^2$ | Irradiated area- $0.5cm^2$/ point ($4cm^2$ total irradiated area) | $8J/cm^2$/point | $400mW/cm^2$/ point | 20 sec/ point | 4 sessions (Once a week) | 4 weeks |
