# Supplementary material for: Role of Photobiomodulation Therapy in Modulating Oxidative Stress in Temporomandibular Disorders. A Systematic Review and Meta-Analysis of Human Randomised Controlled Trials
Source: Antioxidants (Basel). 2021 Jun 25;10(7):1028. doi: 10.3390/antiox10071028 (PMC8300797; doi:10.3390/antiox10071028)
Supplement: Supplementary file 1 [file antioxidants-10-01028-s001.zip › supplementary-2021.7.1/Table S3.pdf]

Table S3. Tabular description of all the selected eligible *in vivo* RCTs human studies of TMD, in terms of level of significance in subjective and objective assessments of pain, functionality improvement and anxiety reduction/QoL improvement. The abbreviations are listed in Supplementary File S2.

Study, Year, Origin and Citation	Pain reduction				Functionality improvement				Anxiety reduction/QoL improvement			
	Qualitative measures (patient self-reporting)		Quantitative measures		Qualitative measures (patient self-reporting)		Quantitative measures		Qualitative measures (patient self-reporting)		Quantitative measures	
	Statistically significant Y/N/NI/NS	Not statistically significant Y/N/NI/NS	Statistically significant Y/N/NI/NS	Not statistically significant Y/N/NI/NS	Statistically significant Y/N/NI/NS	Not statistically significant Y/N/NI/NS	Statistically significant Y/N/NI/NS	Not statistically significant Y/N/NI/NS	Statistically significant Y/N/NI/NS	Not statistically significant Y/N/NI/NS	Statistically significant Y/N/NI/NS	Not statistically significant Y/N/NI/NS
Venancio et al., 2005, Brazil [57]	N	Y	NS	NS	NS	NS	N	Y	NS	NS	NS	NS
Çetiner et al., 2006, Turkey [58]	Y	N	NS	NS	NS	NS	Y	N	NS	NS	NS	NS
Fikackova et al., 2007, Czech Republic [59]	Y	N	NS	NS	NS	NS	NS	NS	NS	NS	NS	NS
Mazzetto et al., 2007, Brazil [60]	Y	N	NS	NS	NS	NS	NS	NS	NS	NS	NS	NS
Frare et al., 2008, Brazil [61]	Y	N	NS	NS	NS	NS	NS	NS	NS	NS	NS	NS
Da Cunha et al., 2008, Brazil [62]	N	Y	NS	NS	NS	NS	N	Y	NS	NS	NS	NS
Lassemi et al., 2008, Iran [63]	Y	N	NS	NS	NS	NS	Y	N	NS	NS	NS	NS
Carrasco et al., 2008, Brazil [64]	Y	N	N	Y	NS	NS	N	Y	NS	NS	NS	NS
Emshoff et al., 2008, Austria [65]	N	Y	NS	NS	NS	NS	NS	NS	NS	NS	NS	NS
Carrasco et al., 2009, Brazil [66]	Y	N	NS	NS	NS	NS	NS	NS	NS	NS	NS	NS
Shirani et al., 2009, Iran [67]	Y	N	NS	NS	NS	NS	NS	NS	NS	NS	NS	NS
Venezian et al., 2010, Brazil [68]	Y	N	NS	NS	NS	NS	Y	N	NS	NS	NS	NS
Oz et al., 2010, Turkey [69]	N	Y	N	Y	NS	NS	N	Y	NS	NS	NS	NS
Marini et al., 2010, Italy [70]	Y	N	NS	NS	NS	NS	Y	N	NS	NS	NS	NS
Rohlig et al., 2011, Turkey [71]	Y	N	Y	N	NS	NS	Y	N	NS	NS	NS	NS
Sattayut et al., 2012, United Kingdom [72]	Y	N	Y	N	NS	NS	Y	N	NS	NS	NS	NS
De Carli et al., 2012, Brazil [73]	N	Y	NS	NS	NS	NS	N	Y	NS	NS	NS	NS
da Silva et al., 2012, Brazil [74]	Y	N	NS	NS	NS	NS	Y	N	NS	NS	NS	NS
Panhoca et al., 2013, Brazil [75]	N	Y	NS	NS	NS	NS	N	Y	NS	NS	NS	NS
Uemoto et al., 2013, Brazil [76]	Y	N	N	Y	NS	NS	N	Y	NS	NS	NS	NS
Ahrari et al., 2014, Iran [77]	N	Y	NS	NS	NS	NS	N	Y	NS	NS	NS	NS
Demirkol et al., 2014, Turkey [78]	N	Y	N	Y	NS	NS	N	Y	NS	NS	NS	NS
Pereira et al., 2014, Brazil [79]	Y	N	NS	NS	NS	NS	NS	NS	Y	N	NS	NS
Maia et al., 2014, Brazil [80]	Y	N	Y	N	NS	NS	Y	N	NS	NS	NS	NS
Sancakli et al 2015, Turkey [81]	Y	N	Y	N	NS	NS	Y	N	NS	NS	NS	NS
De Oliveira et al., 2017, Brazil [82]	NS	NS	NS	NS	NS	NS	N	Y	NS	NS	NS	NS
Costa et al., 2017, Brazil [83]	Y	N	N	Y	NS	NS	NS	NS	NS	NS	NS	NS
Seifi et al., 2017, Iran [84]	Y	N	NS	NS	NS	NS	Y	N	NS	NS	NS	NS
Shobha et al., 2017, India [85]	N	Y	NS	NS	NS	NS	N	Y	NS	NS	NS	NS
Rezazadeh et al., 2017, Iran [86]	Y	N	NS	NS	NS	NS	Y	N	NS	NS	NS	NS
Varma et al., 2018, UAE [87]	Y	N	NS	NS	NS	NS	NS	NS	NS	NS	NS	NS

Borges et al., 2018, Brazil [88]	Y	N	NS	NS	NS	NS	Y	N	NS	NS	NS	NS
Brochado et al., 2018, Brazil [89]	Y	N	NS	NS	NS	NS	Y	N	Y	N	NS	NS
Rodrigues et al., 2018, Brazil [90]	Y	N	NS	NS	NS	NS	NS	NS	NS	NS	NS	NS
Peimani et al., 2018, Iran [91]	Y	N	NS	NS	NS	NS	Y	N	NS	NS	NS	NS
Nadershah et al., 2019, Saudi Arabia [92]	Y	N	NS	NS	NS	NS	NS	NS	NS	NS	NS	NS
Magri et al., 2019, Brazil [93]	Y	N	NS	NS	NS	NS	Y	N	NS	NS	NS	NS
Al-Quisi et al., 2019, Iraq [94]	Y	N	NS	NS	NS	NS	NS	NS	NS	NS	NS	NS
Herpich et al., 2019, Brazil [95]	Y	N	NS	NS	Y	N	N	Y	NS	NS	NS	NS
Khairnar et al., 2019, India [96]	Y	N	NS	NS	NS	NS	Y	N	NS	NS	NS	NS
Sobral et al., 2020, Brazil [97]	Y	N	NS	NS	NS	NS	Y	N	Y	N	NS	NS
Maracci et al., 2020, Brazil [98]	NS	NS	NS	NS	NS	NS	N	Y	N	Y	NS	NS
Chellappa et al., 2020, India [99]	Y	N	NS	NS	NS	NS	NS	NS	NS	NS	NS	NS
Monteiro et al., 2020, Brazil [100]	Y	N	NS	NS	NS	NS	Y	N	NS	NS	NS	NS
